# Altered rhythmic and arrhythmic electroencephalographic activity during non-rapid eye movement sleep in amnestic mild cognitive impairment

**DOI:** 10.1093/braincomms/fcag204

**Published:** 2026-06-22

**Authors:** Alexandre Lafrenière, Jean-Marc Lina, Claire André, Marie-Ève Martineau-Dussault, Dominique Lorrain, Célyne Bastien, Carol Hudon, Nadia Gosselin, Julie Carrier

**Affiliations:** Center for Advanced Research in Sleep Medicine, Hôpital du Sacré-Cœur de Montréal, CIUSSS du Nord-de-l'Île-de-Montréal, Montreal, Canada, H4J 1C5; Department of Psychology, Université de Montréal, Montreal, Canada, H2V 2S9; Center for Advanced Research in Sleep Medicine, Hôpital du Sacré-Cœur de Montréal, CIUSSS du Nord-de-l'Île-de-Montréal, Montreal, Canada, H4J 1C5; Department of Electrical Engineering, École de Technologie Supérieure, Montreal, Canada, H3C 1K3; Centre de Recherches Mathématiques, Université de Montréal, Montreal, Canada, H3T 1N8; Center for Advanced Research in Sleep Medicine, Hôpital du Sacré-Cœur de Montréal, CIUSSS du Nord-de-l'Île-de-Montréal, Montreal, Canada, H4J 1C5; Department of Psychology, Université de Montréal, Montreal, Canada, H2V 2S9; Center for Advanced Research in Sleep Medicine, Hôpital du Sacré-Cœur de Montréal, CIUSSS du Nord-de-l'Île-de-Montréal, Montreal, Canada, H4J 1C5; Department of Psychology, Université de Montréal, Montreal, Canada, H2V 2S9; Research Centre on Aging, University Institute of Geriatrics of Sherbrooke, CIUSSS de l'Estrie—CHUS, Sherbrooke, Canada, J1H 4C4; Department of Psychology, Université de Sherbrooke, Sherbrooke, Canada, J1K 2R1; CERVO Research Centre, Quebec City, Canada, G1E 0N5; School of Psychology, Université Laval, Quebec City, Canada, G1V 0A6; CERVO Research Centre, Quebec City, Canada, G1E 0N5; School of Psychology, Université Laval, Quebec City, Canada, G1V 0A6; Center for Advanced Research in Sleep Medicine, Hôpital du Sacré-Cœur de Montréal, CIUSSS du Nord-de-l'Île-de-Montréal, Montreal, Canada, H4J 1C5; Department of Psychology, Université de Montréal, Montreal, Canada, H2V 2S9; Center for Advanced Research in Sleep Medicine, Hôpital du Sacré-Cœur de Montréal, CIUSSS du Nord-de-l'Île-de-Montréal, Montreal, Canada, H4J 1C5; Department of Psychology, Université de Montréal, Montreal, Canada, H2V 2S9

**Keywords:** Alzheimer’s disease, sleep, slow-wave sleep, electroencephalography, mild cognitive impairment

## Abstract

Non-rapid eye movement sleep electroencephalographic activity has been proposed to provide helpful markers for the early detection of Alzheimer’s disease. However, studies have produced mixed results regarding the impact of mild cognitive impairment—a prodromal stage—when using traditional spectral power analyses during this sleep phase. It is increasingly recognized that electrophysiological power spectra are composed of two components: a rhythmic part, which reflects oscillatory activity, and an arrhythmic part, which represents the balance of neuronal excitation and inhibition. In this cross-sectional study, our objective was thus to determine whether cognitive impairment is associated with the non-rapid eye movement sleep rhythmic and arrhythmic components in older adults. Fifty-six cognitively normal participants (68.3 ± 7.1 years old, 41% women) and 56 participants with amnestic mild cognitive impairment (69.9 ± 8.6 years old, 41% women) matched on multiple variables (age, sex, education, body mass index, apnoea–hypopnoea index) underwent a neuropsychological assessment and a night of polysomnography. Analyses focused on six channels of interest (F3, F4, C3, C4, P3 and P4). Significant between-group differences were observed for non-rapid eye movement sleep rhythmic and arrhythmic activity. Participants with amnestic mild cognitive impairment had lower rhythmic power in the fast-sigma/slow-beta range across all topographies investigated and higher gamma rhythmic power in the left parietal area. Participants with amnestic mild cognitive impairment also showed lower mean gamma aperiodic exponents—an arrhythmic activity measure—across all topographies. Frontal rhythmic power in the fast-sigma/slow-beta range predicted episodic memory in the amnestic mild cognitive impairment group. Our findings reveal alterations in both rhythmic and arrhythmic brain activity, indicating oscillatory changes and signs of hyperexcitability during non-rapid eye movement sleep in individuals with amnestic mild cognitive impairment.

## Teaser

Lafrenière *et al*. report that sleep EEG spectral decomposition reveals lower fast-sigma/slow-beta rhythmic power, higher gamma rhythmic power and altered gamma arrhythmic activity during non-rapid eye movement sleep in individuals with amnestic mild cognitive impairment, suggesting disrupted thalamo-cortical regulation and increased neuronal hyperexcitability associated with cognitive decline.

## Graphical Abstract

**Graphical Abstract:**
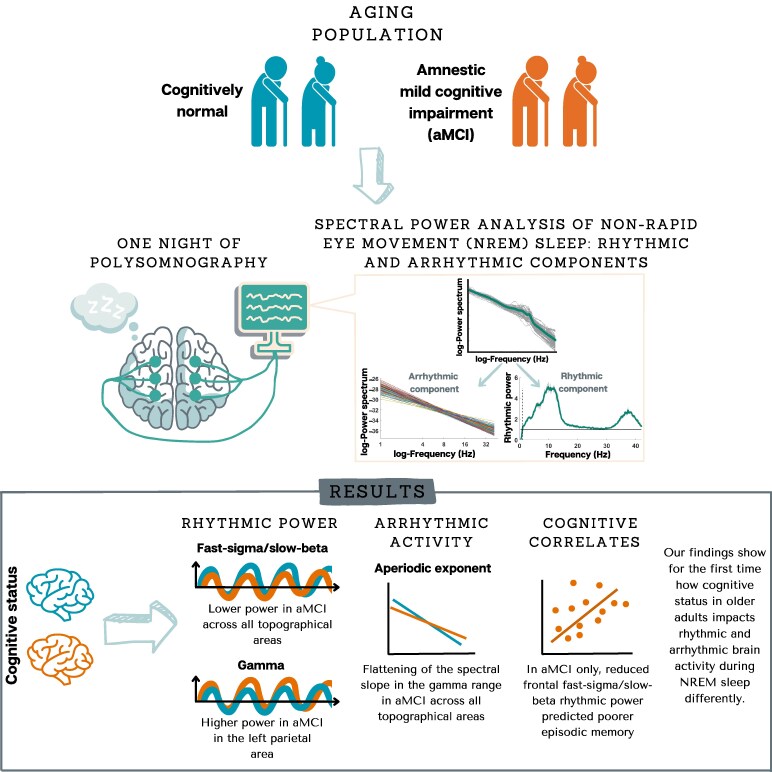

## Introduction

Alzheimer’s disease is the most prevalent cause of dementia and a worldwide public health priority.^1,2^ The neurodegenerative process of Alzheimer’s disease begins several years before the onset of dementia.^3^ Amnestic mild cognitive impairment (aMCI) represents a transitory stage between normal cognitive ageing and prototypical Alzheimer’s disease dementia.^4,5^ This prodromal stage offers the opportunity to identify early brain changes in individuals at higher risk of progressing to Alzheimer’s disease.^5-10^ In this context, monitoring sleep changes has been proposed to provide valuable markers relevant to tracking the onset of Alzheimer’s disease dementia.^11,12^

Non-rapid eye movement (NREM) sleep in humans is divided into three stages (i.e. N1–N3), coinciding with a progressive deepening of sleep mirrored in brain oscillatory activity.^13^ NREM sleep electroencephalographic activity has been associated with cognitive functioning across the human life continuum.^14-16^ Furthermore, this cerebral activity is theorized to underpin sleep-dependent neuroplasticity processes,^17^ with a central role for oscillatory events, such as slow waves (SWs) (delta spectral range: 0.25–4 Hz) and sleep spindles (sigma spectral range: 10–16 Hz). A traditional way to probe EEG changes during sleep is to perform spectral power analysis,^18^ which describes the signal’s energy distribution in the frequency domain. Throughout adulthood, noticeable age-related changes in spectral activity are observable during NREM sleep.^19,20^ Compared to younger adults, older adults show lower spectral power in the delta and sigma activity ranges, with some studies also indicating higher power in the beta/gamma range.^21-28^ During healthy ageing, inter-individual differences of spectral power in the delta band have also shown associations with current^16^ and longitudinal cognitive performance,^29^ current^30,31^ and future amyloid-β pathology^32^ and tau pathology.^33^ Similarly, sigma band power has been associated with cognitive performance in older individuals.^16,34^ Moreover, it has recently been shown in a mediation model that glial activation, via an effect on tau-related and synaptic integrity biomarkers, was related to deficits in fast-sigma spectral activity in normocognitive older individuals with enriched Alzheimer’s disease–related medical history.^35^ Altogether, these studies suggest that changes in spectral activity during NREM sleep may reflect early pathological processes in normal ageing, before the onset of cognitive deficits.

Despite the accumulating evidence linking NREM sleep brain activity and cognitive functioning in normal ageing,^16,29,36-38^ few studies have investigated NREM sleep spectral power changes in the context of MCI.^39,40^ Even fewer studies examined these changes specifically in aMCI. Moreover, those studies have offered mixed results, and no consistent patterns of alterations have been identified so far.^41-45^ For example, one study^43^ reported lower delta power, whereas two studies found no differences,^41,44^ between older adults with and without MCI. Among those same studies, Westerberg *et al*.^43^ reported lower theta power and no differences in sigma power in older adults with MCI. Conversely, D'Atri *et al*.^41^ observed lower sigma power and no differences in theta power in MCI. No differences were observed in alpha^41,43^ and beta^41^ power between groups in those studies. For the NREM sleep of a daytime nap, no difference in delta and sigma power was reported between older adults with and without MCI.^45^ Although these mixed results might have arisen from heterogeneity in sample characteristics, experimental protocols or the topography examined, they could have emerged due to the method by which spectral power changes were probed.

It is increasingly recognized that electrophysiological power spectra are composed of two components: a periodic/rhythmic and an aperiodic/arrhythmic part.^46^ The periodic component refers to oscillatory neuronal activity and manifests as peaks over the background of aperiodic fluctuations.^47^ Physiologically, neuronal oscillations have been proposed to reflect the synchronization of neuron activity within neural circuits.^48-50^ The aperiodic component of the EEG signal follows a $1/f^{\beta }$ power law, reflecting the regular decay in power as frequency increases across the spectrum.^46,51,52^ This aperiodic activity can be quantified using different parameters, one of which is the spectral slope (*−β*), also known as the ‘aperiodic exponent’, as it corresponds to the scaling exponent *β* in the power law function.^51,52^ The gamma-range spectral slope (≈30–50 Hz) has been proposed as an index of the excitatory–inhibitory (E–I) balance of brain networks, reflecting synaptic current integration and background neural dynamics.^53-55^ Increased inhibitory activity translates as a steeper spectral slope in this frequency range. Moreover, the gamma spectral slope has been shown as a cortical arousal marker, where the slope becomes progressively steeper across wakefulness, NREM sleep and REM sleep, respectively.^56,57^ Recent studies have also reported that the global broadband spectral slope (2–48 Hz range) measured during NREM sleep underwent an age-related flattening.^58,59^ In this regard, neuronal hyperexcitability resulting from an E–I imbalance has been proposed to underlie brain network dysfunction and cognitive deficits early in the Alzheimer’s disease spectrum.^60^ Moreover, MCI is consistently related to more wake after sleep onset, reduced sleep efficiency and more N1 sleep stage, all reflecting a higher degree of arousal across sleep.^40^ Therefore, this potential shift towards increased neuronal excitability^61-63^ and shallower sleep in MCI could be captured by the gamma aperiodic exponent during NREM sleep.

Failing to account for the spectral slope’s influence over the EEG power spectrum can obscure classical spectral power estimates.^49,51^ Considering the rhythmic and arrhythmic EEG components could also provide insight into whether these spectral components are sensitive to early brain changes associated with cognitive impairment in ageing. Therefore, we first compared changes in NREM sleep EEG power between older adults with and without aMCI, using classical absolute spectral power estimates. We expected power reductions in the delta, theta and sigma spectral ranges in the aMCI group. Then, we investigated group differences in the rhythmic and arrhythmic components of NREM sleep. We predicted a decrease in rhythmic power in the delta and sigma frequency ranges, as well as lower gamma and broadband aperiodic exponent values (indicating flatter slopes) in individuals with aMCI. Finally, we predicted that alterations in rhythmic and arrhythmic EEG components would be associated with composite memory and executive function performance scores, with stronger associations observed in the aMCI group.

## Materials and methods

### Participants

This cross-sectional study included a total of 56 cognitively normal (CN) participants and 56 participants with aMCI who were matched for age, sex, education, body mass index (BMI) and the apnoea–hypopnoea index (AHI; see Supplementary Fig. 1). Participants’ ages ranged from 56 to 85 years old. Participants’ data came from four research protocols approved by institutional ethics committees (#2012-697, #2011-690, #2010-468 and #MP-32-2018-1537) between 2012 and 2020. From the total sample (*N* = 112), 75 participants (*n* = 38 CN; *n* = 37 aMCI) were recruited in Montreal as part of three protocols investigating sleep, ageing and cognition. Thirty-seven participants (*n* = 18 CN; *n* = 19 aMCI) were recruited from three sites (Montreal, Sherbrooke and Quebec City) as part of a provincial multicentric project on sleep and MCI. Portions of the present dataset have been included in previously published studies.^64-66^ All participants provided written informed consent in accordance with the Declaration of Helsinki and were recruited through local memory and sleep apnoea clinics, previous research protocols or advertisements in local newspapers.

Exclusion criteria were as follows: <7 years of education, presence or history of neurological disorders (e.g. epilepsy, traumatic brain injury, stroke and encephalopathy), current psychiatric disorders based on DSM-V criteria (e.g. dementia, diagnosis of anxiety disorders or depressive disorders), sleep disorders (e.g. insomnia, narcolepsy, restless legs syndrome, REM sleep behaviour disorder and treated obstructive sleep apnoea), pulmonary diseases (e.g. chronic obstructive pulmonary disease), uncontrolled diabetes or hypertension, BMI > 40 kg/m^2^ and use of psychotropic substances (including illicit drugs or alcohol addiction or abuse) that can impact sleep, brain functions or cognition (e.g. antidepressants and hypnotics). Of note, no participants had a diagnosis of obstructive sleep apnoea (OSA) at the start of the study. However, some participants had AHI > 15 during the polysomnographic (PSG) recording. Therefore, we matched our groups based on AHI in this study.

All participants were screened by phone for potential eligibility and then scheduled for an in-person interview, a complete neuropsychological assessment and one full-night in-laboratory PSG recording.

### Neuropsychological assessment and cognitive composite scores

Five cognitive domains were evaluated: attention and processing speed, executive functions, episodic learning and memory, visuospatial abilities and language. Although some neuropsychological tests differed between protocols, a set of core tests was available for all participants for every cognitive domain (see Supplementary Table 1). The participants’ cognitive status was determined by clinical neuropsychologists based on all available cognitive tests. More specifically, the aMCI diagnosis was based on four criteria^5,67^: (i) subjective cognitive complaints linked to cognitive changes in the last 6 months assessed through questionnaires; (ii) an objective cognitive impairment defined as a *Z*-score ≤ 1.5 SD below normative data on at least two measures in the memory domain (alone or with coexistent impairment in other cognitive domains); (iii) preservation of independence in daily activities as evidenced by a questionnaire or during the case history interview; and (iv) absence of dementia and the cognitive impairment not better explained by a medical or psychiatric condition or medication use. Of note, some of these criteria were assessed differently across the protocols (for more details, see Supplementary Table 2). CN participants had to be exempt from objective cognitive impairment in all cognitive domains and scored ≥26 on the Montreal Cognitive Assessment (MoCA) test. Finally, cognitive composite scores were computed for episodic memory and executive functions. The procedure to calculate these composite scores was inspired by Perrotin *et al*.’s^68^ methodology and based on our previous work^69^ (see Supplementary material, Methods section).

### Questionnaires

All participants completed questionnaires evaluating excessive daytime sleepiness, anxiety and depressive symptoms. The former was assessed using the Epworth Sleepiness Scale (ESS).^70^ As there were variations in the questionnaires used to assess anxiety and depressive symptoms across the protocols, a dichotomization of the measures’ scores was performed using established cut-offs. The presence of significant anxiety symptoms was determined by a score of ≥8 on the Beck Anxiety Inventory^71^ and the Geriatric Anxiety Inventory.^72,73^ The presence of significant depressive symptoms was determined by a score of ≥14 on the Beck Depression Inventory II^74^ and ≥ 5 on the Geriatric Depression Scale.^75^

### Polysomnographic recording and scoring

The PSG recording was conducted in a sleep laboratory, comprising a 12–19-channel EEG montage with placement based on the international 10–20 system and used mastoid references. Seventy-nine participants’ sleep (*n* = 42 CN; *n* = 37 aMCI) was recorded with a Grass system (bandpass filter 0.3–100 Hz; hereon termed ‘System 1’) with the signal digitized at a sampling rate of 256 Hz using commercial software (Harmonie, Stellate Systems). Thirty-three participants’ sleep (*n* = 14 CN; *n* = 19 aMCI) was recorded with a Natus system (Brain Monitor, Trex or Embla NDx; bandpass filter 0.3–200 Hz; hereon termed ‘System 2’) with the signal digitized at a sampling rate of 512 Hz. Of note, there was no statistically significant difference between the aMCI and CN groups regarding the prevalence of the EEG system utilized (Fisher’s exact test, *P* = 0.407). The PSG also included an electrooculogram, an electrocardiogram and a submental electromyogram. A bilateral anterior tibialis muscle electromyogram was utilized to measure periodic leg movements. Monitoring respiration was realized via thoracoabdominal strain gauges, oronasal thermistors and oronasal cannula. Oxygen saturation was measured via a transcutaneous finger pulse oximeter.

Based on standard methods,^76,77^ the recording and visual scoring of sleep in 30-s epochs were achieved by experienced medical electrophysiology technologists at the Montreal site. Episodes of sleep apnoeas were defined as a reduction of ≥90% from baseline airflow, lasting ≥10 s. Episodes of sleep hypopnoeas were defined as a drop in airflow amplitude of ≥30% from baseline for ≥10 s, accompanied either by an oxygen desaturation of ≥3% or by an EEG arousal. The AHI was computed as the sum of obstructive apnoeas and hypopnoeas divided by the total number of hours of sleep.

### Preprocessing of the EEG data

First, EEG signals were processed using an automatic muscle artefact detection algorithm,^78^ followed by manual review by a trained technologist. During visual inspection, residual artefacts, including muscle activity, ocular movements, cardiac artefacts and 60 Hz line noise, were identified and excluded from subsequent analyses. Persistent 60 Hz line noise was removed with a notch filter, whereas intermittent contamination was manually rejected. All spectral power analyses were performed on artefact-free epochs of combined N2 and N3 sleep (hereafter referred to as NREM sleep) using six electrodes of interest (F3, F4, C3, C4, P3 and P4). These channels were selected because they were consistently available across all participants, thereby maximizing sample size while avoiding the need for signal interpolation. Raw recordings in the EDF format were imported in MATLAB (MathWorks R2018b) using custom scripts to create the final artefact-free 30 s epochs and conduct the spectral power analyses. EEG data were down-sampled to 200 Hz prior to spectral analyses.

### Absolute spectral power analysis

Power spectra were computed using a fast Fourier transform (FFT) applied to 30-s epochs, resulting in a frequency resolution of $\frac{1}{30}$ Hz (∼0.033 Hz). Squared Fourier amplitudes were calculated for frequencies between 1 and 42 Hz, and absolute spectral power per frequency (μV^2^/Hz) was estimated for each epoch and subsequently averaged at the subject level. No additional tapering window was applied. Specifically, the power spectrum of each epoch was denoted by $p_{i}(f)$, with ‘*i*’ representing the epoch. For each subject, the mean power spectrum during NREM sleep was computed as:


(1) $$P_{I}(f)=\;\frac{1}{n_{I}}\;\underset{i\in I}{\sum }\;p_{i}\;(f)$$

where *I* denotes the set of all NREM sleep epochs (combining N2 and N3) and $n_{I}$ is the total number of epochs included in the average.

### Spectral power analysis of rhythmic and arrhythmic components

In this study, NREM rhythmic power was estimated using a multiplicative spectral modelling approach,^58^ which has been extensively validated in sleep EEG research. This framework has been successfully applied to animal models,^79^ intracranial recordings,^80^ healthy human samples across adulthood^58,81,82^ and clinical populations.^83,84^ This multiplicative model was applied to each $n_{I}$ artefact-free epoch:


(2) $$p_{i}(f)=\;\frac{c_{i}}{\;f^{\beta_{i}\;}}\;e^{r_{i}(f)},\;\;\;\;\;\;\;i=1,2,\ldots ,\;n_{I}$$

**Figure 1:**
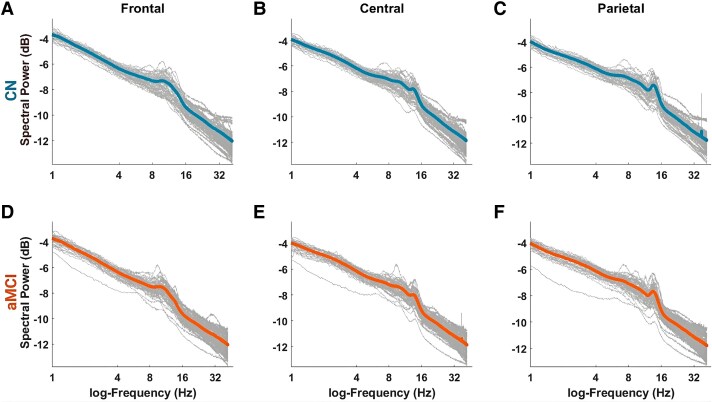
**Log-transformed EEG power spectra illustrating the linear spectral organization across groups and topographies.** Thick coloured lines show group-averaged power spectra (CN, *N* = 56; aMCI, *N* = 56), while thin grey lines represent participant-level mean spectra. Panels depict frontal (**A** and **D**), central (**B** and **E**) and parietal (**C** and **F**) regions for the CN (**A–C**) and aMCI (**D–F**) groups. dB, decibels.

**Figure 2:**
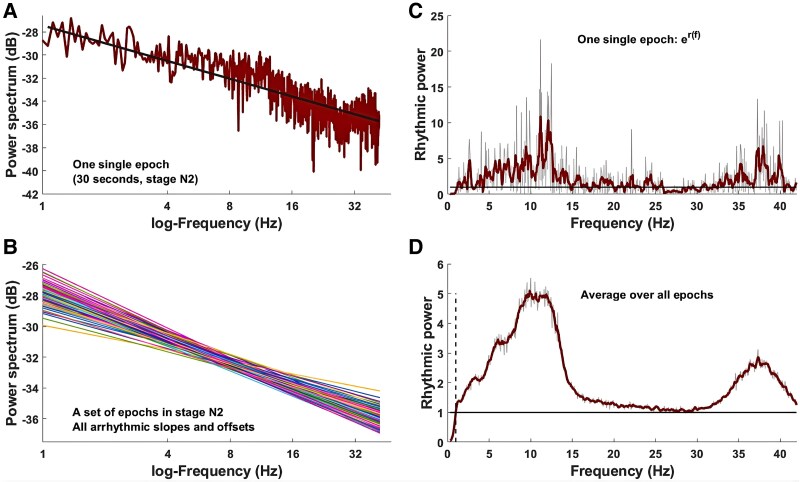
**Example of EEG power spectrum decomposition into rhythmic and arrhythmic components in a single participant.** (**A**) Power spectral density from one N2 epoch (*N* = 1), with the dark straight line indicating the fitted spectral slope. (**B**) Spectral slopes (1–42 Hz) and corresponding offsets derived across multiple N2 epochs, illustrating the temporal variability of arrhythmic activity. (**C**) Rhythmic power estimated from a single epoch. (**D**) Rhythmic power averaged across all artefact-free epochs. dB, decibels.

In this formulation, the arrhythmic component is related to the $\frac{c_{i}}{\;f^{\beta_{i}\;}}$ part, and the rhythmic component is described by the exponential factor $e^{r_{i}(f)}$. In more detail, when the power and frequency axis are log transformed, most of the EEG spectrum becomes characterized by a dominant broadband linear pattern. Figure 1 illustrates this pattern. This linear spectral organization emerging in log–log scales translate the so-called temporal scale-free activity, which can be modelled with a power law function described by $1/f^{\beta_{i}}$. Up to a negative sign, the exponent $\beta_{i}\;$ is the slope of the spectra. Accordingly, the aperiodic exponent is conventionally reported as a positive-valued parameter, whereas the spectral slope refers to the same parameter expressed with a negative sign: lower exponent values indicate a flatter (i.e. less negative) underlying spectral slope, whereas higher values reflect a steeper (more negative) slope. Figure 2A’s dark line exemplifies this slope for one participant in one epoch. Importantly, this slope, and therefore the exponent, may vary across epochs throughout the night, reflecting the dynamical property of this scale-free, arrhythmic activity (see Fig. 2B). For this reason, and as shown in Eq. 3, we estimated the parameters for each NREM sleep epoch. More precisely, we estimated the offset $c_{i}^{*}$ and the scaling exponent $\beta_{i}^{*}$ from the linear regression of the power spectrum $p_{i}$ in the log–log space. For group comparison analyses, we estimated the *β* exponent (slope), hereon termed ‘aperiodic exponent’, for two specific spectral ranges: broadband (1–42 Hz) and gamma (30–42 Hz). We subsequently estimated the rhythmic component from the interval *I* corresponding to all NREM sleep epochs. This component was measured via the ‘residual spectral power’ R(f), which was computed as follows:


(3) $$e^{R^{*}(f)}=\;\frac{1}{n_{I}}\;\underset{i\in I}{\sum }\frac{\;f^{\beta_{i}^{*}}}{c_{i}^{*}}\;p_{i}\;(f)$$

**Figure 3:**
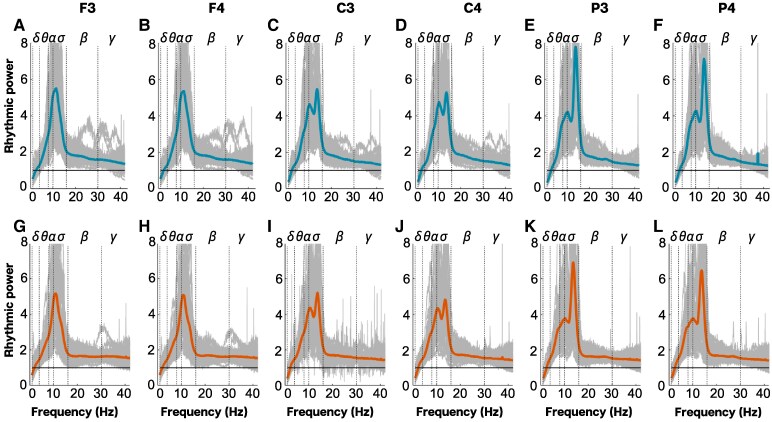
**Distribution of rhythmic power across the frequency spectrum by cognitive group and topography.** Thick coloured lines depict group-averaged rhythmic power spectra (1–42 Hz) for CN (blue; *N* = 56; panels **A–F**) and aMCI (orange; *N* = 56; panels **G–L**). Grey thin lines represent participant-level mean rhythmic power.

The residual or ‘rhythmic’ spectral power of one participant in one epoch is illustrated in Fig. 2C, and the average over all epochs in Fig. 2D. Figure 3 displays the distribution of rhythmic power specific to each group and each topographical area. Of note, the lower frequency range of the spectrum (i.e. 0.3–1 Hz) can be influenced by the recording device’s high-pass filter and the EEG system’s model (see Supplementary Fig. 2). More specifically, a ‘spectral knee’ towards the slower frequencies creates a ‘spectral plateau’. Thus, the spectral slope does not fit properly in this region, hampering the estimation of the superimposed rhythmic activity. This spectral plateau can also have a different tilt depending on the EEG system. For these reasons, the EEG activity in this spectral range is not well captured in the abovementioned procedure. Consequently, we performed a separate analysis to estimate the rhythmic spectral power in the slow-delta range. We used the same method as described above but specifically applied to the 0.3–1 Hz frequency range (for more details, see Supplementary Fig. 3). As the *β* exponent (slope) considers the spectral plateau’s tilt, this enabled us to overcome the potential differences secondary to the EEG system used when estimating the rhythmic power in this spectral range (see Supplementary Fig. 4).

### Cluster-based permutation testing

Non-parametric cluster-based permutation testing assessed differences between the aMCI and CN groups on absolute and rhythmic spectral power.^85^ The procedure was as follows: (i) participants’ datasets from the aMCI and CN groups were pooled together to form a single set; (ii) participants’ datasets were randomly shuffled into two temporary subsets disregarding original group labels, creating a random partition; (iii) using Eq. 4, an independent-samples *t*-test was calculated at each frequency (*f*) of the power spectrum for each random partition:


(4) $$t(f)=\;\frac{\mu_{A}(f)-\mu_{B}(f)}{\sqrt{\frac{\sigma_{A}^{2}}{N_{A}}+\frac{\sigma_{B}^{2}}{N_{B}}}}$$

(iv) from each random partition, clusters were identified when three contiguous *t*-values or more bearing the same sign were superior to a *t*-value of ±2. This cluster-forming threshold corresponds to the critical value of a bilateral *t*-test at alpha-level 0.05 in the *t*-distribution; (v) for each random partition, cluster-level statistics were calculated by taking the sum of the *t*-values within a cluster (delineated by the threshold in step 4); (vi) the largest of the cluster-level statistic was collected from each random partition and stored; (vii) an iteration of Steps 2–6 was repeated 5000 times to generate a null distribution (*H_0_*) of possible, randomly obtained largest cluster-level statistics; and (viii) clusters resulting from real groups’ comparisons were finally identified (as in Step 4), and their respective *P* value was calculated under the permuted, null distribution (*H_0_*) generated in Step 7. By forming clusters within the high-dimensional EEG dataset, this approach permits a control for Type I error rate due to multiple comparisons.^86^ For these analyses, clusters were considered significant when reaching a bilateral significance level of *P* < 0.05, corresponding to each permuted, null distribution’s 2.5th and 97.5th percentile.

### Statistical analyses

Variables for parametric analyses were examined for normality and homogeneity of variance using measures of skewness and kurtosis, the Shapiro–Wilk test, visual inspection of standardized residual plots and the *F*_max_ test.^87,88^ A deviation from normality was corrected via natural log (*Ln*) transformation (only applied to absolute spectral power estimates) if no non-parametric test alternative was available. Outliers were identified as values at ±3.29 SDs from the mean and were winsorized. For correlational analyses, bivariate outliers were identified using a standardized residual score of >2 SD and Cook’s distance to evaluate each data point’s influence level.

For the characterization of our groups, independent-sample *t*-tests or Mann–Whitney U-tests were performed for sociodemographics and PSG variables. Fisher’s exact tests were carried out to assess group differences in sex, anxiety and depressive symptom prevalence. As we were interested in the aperiodic exponent values covering specific frequency ranges (i.e. we had no interest in the value at each frequency) and the slow-delta range (0.3–1 Hz) did not include enough points within its spectral interval, cluster-based permutation testing could not be used. Consequently, to evaluate group differences on the aperiodic exponents and the rhythmic spectral power in the slow-delta range, two-way mixed ANOVAs with one independent factor (cognitive status) and one repeated measure (topographical regions) were conducted. For these analyses, rhythmic and arrhythmic measures were averaged over topographical areas to reduce the number of comparisons: frontal (average of F3 and F4), central (average of C3 and C4) and parietal (average of P3 and P4). According to Girden’s^89^ recommendations, *P* values for repeated measures with more than two levels were adjusted for sphericity: when ε > 0.75, the Huynh–Feldt correction was applied; when ε < 0.75, the Greenhouse–Geisser correction was applied. Effect sizes were estimated via the partial eta^2^ (η^2^*p*) and reported for significant effects for all ANOVAs. Finally, we examined whether the associations between EEG spectral measures showing group differences and cognitive performance differed as a function of cognitive status using moderation analyses. Specifically, multiple linear regression models were fitted in which cognitive composite scores (episodic and executive composite scores) served as the dependent variables, the spectral measure and cognitive status were entered as predictors and a spectral measure × cognitive status interaction term was included. These moderation analyses were conducted using the PROCESS macro for SPSS (version 5.0).^90^ Continuous predictor variables were mean centred prior to analysis. Moderation effects were evaluated by testing the statistical significance of the interaction term and the additional variance explained by this interaction. All models included an EEG recording system as a covariate to control for potential system-related effects. Overall, seven separate models were estimated for each cognitive composite score. The significance threshold for all statistical analyses was set at *P* < 0.05 (two-tailed). All statistical analyses were performed using IBM Statistical Packages for Social Sciences v30.0.0.0 (SPSS, 2025), and figures were produced using GraphPad Prism v10 (2023) for Windows and JASP 0.17.2.1 (2023).

### Assessment of the EEG system effect

Verifications were undertaken to determine whether an EEG system effect influenced our results. Thus, we conducted two-way ANOVAs with two independent factors (cognitive status and EEG system) to test the EEG system effect (main and interaction effects) on clusters showing significant between-group differences in absolute and rhythmic power with the permutation analyses. We used the integrated power (i.e. the area under the curve) derived from the significant clusters for these analyses. Three-way mixed ANOVAs with two independent factors (cognitive status and EEG system) and one repeated measure (topographical regions) were performed for the aperiodic exponents and the rhythmic spectral power in the slow-delta range. Finally, as a last precaution, we compared our groups using cluster-based permutation tests and parametric analyses (for the aperiodic exponents and the rhythmic spectral power in the slow-delta range) split according to the EEG system (for more details, see Supplementary material, Methods section). This aimed to verify if the same relative pattern of differences was found between our groups, regardless of the EEG system used. Note, however, that this last set of analyses mainly served descriptive purposes and is only presented in the Supplementary material.

## Results

Characteristics for the aMCI and the CN groups are presented in Table 1. Globally, the two groups were not significantly different on sociodemographic and clinical variables, except for the MoCA score (CN > aMCI), as expected. Regarding sleep architecture, the two groups did not significantly differ on all NREM sleep stages (%) and the AHI. However, compared to the CN group, the aMCI group had significantly shorter sleep duration, reduced sleep efficiency and REM sleep (%) and a higher proportion of wakefulness during the sleep period.

**Table 1:** Group sociodemographics and polysomnographic variables

Sociodemographics	CN *n* = 56	aMCI *n* = 56	Test statistic	Sig.
Age (years)	68.30 (7.13)	69.91 (8.62)	−1.08	0.29
Sex (M/F)^a^	33/23	33/23		1.00
Education (years)	14.98 (3.05)	14.20 (3.50)	1.27	0.21
BMI (kg/m^2^)	26.55 (3.95)	26.41 (3.40)	0.21	0.84
Anxiety symptoms (% with)^a^	10.71	19.64		0.29
Depressive symptoms (% with)^a^	7.14	17.86		0.15
ESS	7.05 (4.41)	7.45 (3.97)	−0.50	0.62
MoCA^b^	28.39 (1.27)	25.04 (2.75)	8.01	**<0.001**
PSG variables				
AHI (events/h)^c^	10.30 (12.60)	10.90 (15.68)	1574.50	0.97
Total sleep time (min.)	366.25 (64.75)	341.24 (64.66)	2.05	**0.043**
Sleep efficiency (%)^c^	81.60 (12.00)	76.70 (15.30)	1218.50	**0.042**
Wake (%)^c^	17.85 (12.33)	23.30 (15.10)	1911.50	**0.046**
N1 stage (%)	17.90 (7.05)	21.13 (10.50)	−1.91	0.059
N2 stage (%)	54.96 (8.77)	56.02 (9.56)	−0.61	0.54
N3 stage (%)^c^	6.95 (16.05)	5.10 (10.65)	1452.50	0.50
REM stage (%)	16.85 (5.46)	14.45 (5.94)	2.22	**0.028**

*Notes.* Independent-sample *t*-tests were used unless stated otherwise. Bold-coloured results identify significant results at *P* < 0.05. Continuous descriptive variables are presented as mean (SD) unless otherwise indicated.

AHI, apnoea–hypopnoea index; BMI, body mass index; ESS, Epworth Sleepiness Scale; MoCA, Montreal Cognitive Assessment.

^a^Fisher’s exact test was used.

^b^Missing data for six participants (*n* = 2 CN, *n* = 4 aMCI).

^c^Mann–Whitney U-test was used, and descriptive variables are displayed as median (interquartile range).

### Absolute spectral power

**Figure 4:**
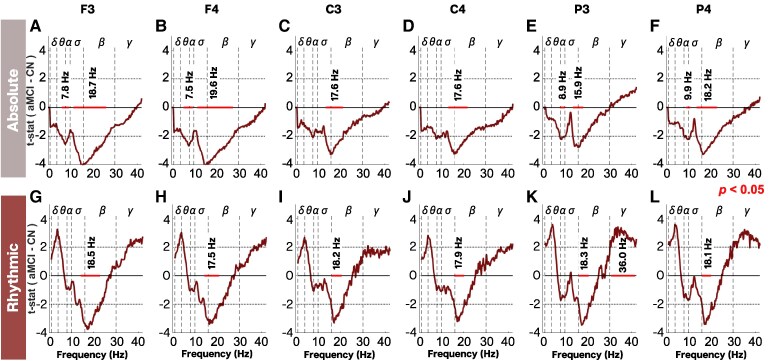
**Significant spectral clusters identified by cluster-based permutation testing in group comparisons.** (**A–F**) Plots of frequency-specific *t*-values derived from non-parametric cluster-based permutation tests for traditional absolute spectral power analyses across topographies (F3: 6.25–9.38 Hz, *t*_max_ = −2.55, *P* < 0.001; F3: 11.38–26.07 Hz, *t*_max_ = −4.06, *P* < 0.001; F4: 5.37–9.57 Hz, *t*_max_ = −2.67, *P* < 0.001; F4: 11.57–27.59 Hz, *t*_max_ = −4.14, *P* < 0.001; C3: 13.67–21.44 Hz, *t*_max_ = −3.31, *P* < 0.001; C4: 13.23–21.88 Hz, *t*_max_ = −3.24, *P* < 0.001; P3: 7.76–10.01 Hz, *t*_max_ = −2.25, *P* = 0.004; P3: 13.57–18.21 Hz, *t*_max_ = −2.82, *P* < 0.001; P4: 8.94–10.84 Hz, *t*_max_ = −2.25, *P* = 0.007; P4: 13.57–22.80 Hz, *t*_max_ = −3.31, *P* < 0.001). (**G–L**) Plots of frequency-specific *t*-values derived from non-parametric cluster-based permutation tests for rhythmic spectral power analyses across the same topographies (F3: 14.16–22.85 Hz, *t*_max_ = −3.72, *P* < 0.001; F4: 14.21–20.85 Hz, *t*_max_ = −3.31, *P* < 0.001; C3: 15.82–20.56 Hz, *t*_max_ = −3.29, *P* < 0.001; C4: 15.72–20.17 Hz, *t*_max_ = −3.19, *P* < 0.001; P3: 16.06–20.61 Hz, *t*_max_ = −3.40, *P* < 0.001; P3: 30.71–41.36 Hz, *t*_max_ = 3.41, *P* = 0.006; P4: 16.06–20.17 Hz, *t*_max_ = −3.40, *P* < 0.001). Thick red lines indicate the frequency range of significant clusters, with numbers above denoting cluster median frequencies. *t*_max_ represents the peak *t*-value within the significant cluster. *N* = 112. *t*-stat = *t*-statistic.

Figure 4 (top row) depicts differences between the aMCI and the CN groups regarding absolute spectral power. Two significant clusters were identified for the frontal area, one covering the theta/alpha range (F3: 6.25–9.38 Hz, *P* < 0.001; F4: 5.37–9.57 Hz, *P* < 0.001) and the other the sigma/beta range (F3: 11.38–26.07 Hz, *P* < 0.001; F4: 11.57–27.59 Hz, *P* < 0.001). One significant cluster was identified for the central area, which covers the fast-sigma/slow-beta range (C3: 13.67–21.44 Hz, *P* < 0.001; C4: 13.23–21.88 Hz, *P* < 0.001). At last, two significant clusters were detected for the parietal area, one covering the theta/alpha range (P3: 7.76–10.01 Hz, *P* = 0.004; P4: 8.94–10.84 Hz, *P* = 0.007) and the other the fast-sigma/slow-beta range (P3: 13.57–18.21 Hz, *P* < 0.001; P4: 13.57–22.80 Hz, *P* < 0.001). For all these clusters, the aMCI group showed significant lower absolute power compared to the CN group.

In sum, the aMCI group exhibited significantly lower absolute spectral power in the theta/alpha and sigma/beta bands in the frontal, central and parietal regions compared to the CN group.

### Rhythmic spectral power

Figure 4 (bottom row) presents group differences regarding rhythmic spectral power. From there, one significant cluster was identified in the frontal area, which covers the fast-sigma/slow-beta spectral range (F3: 14.16–22.85 Hz, *P* < 0.001; F4: 14.21–20.85 Hz, *P* < 0.001). One significant cluster was also detected in the central area, covering the fast-sigma/slow-beta spectral range (C3: 15.82–20.56 Hz, *P* < 0.001; C4: 15.72–20.17 Hz, *P* < 0.001). Two significant clusters were identified for the parietal area, one covering the slow-beta spectral range (P3: 16.06–20.61 Hz, *P* < 0.001; P4: 16.06–20.17 Hz, *P* < 0.001) and the other unilaterally covering the gamma range (P3: 30.71–41.36 Hz, *P* = 0.006). For all significant fast-sigma/slow-beta clusters, the aMCI group showed lower rhythmic power compared to the CN group. Conversely, the aMCI group had significantly higher rhythmic gamma power in the left parietal area compared to the CN group.

**Figure 5:**
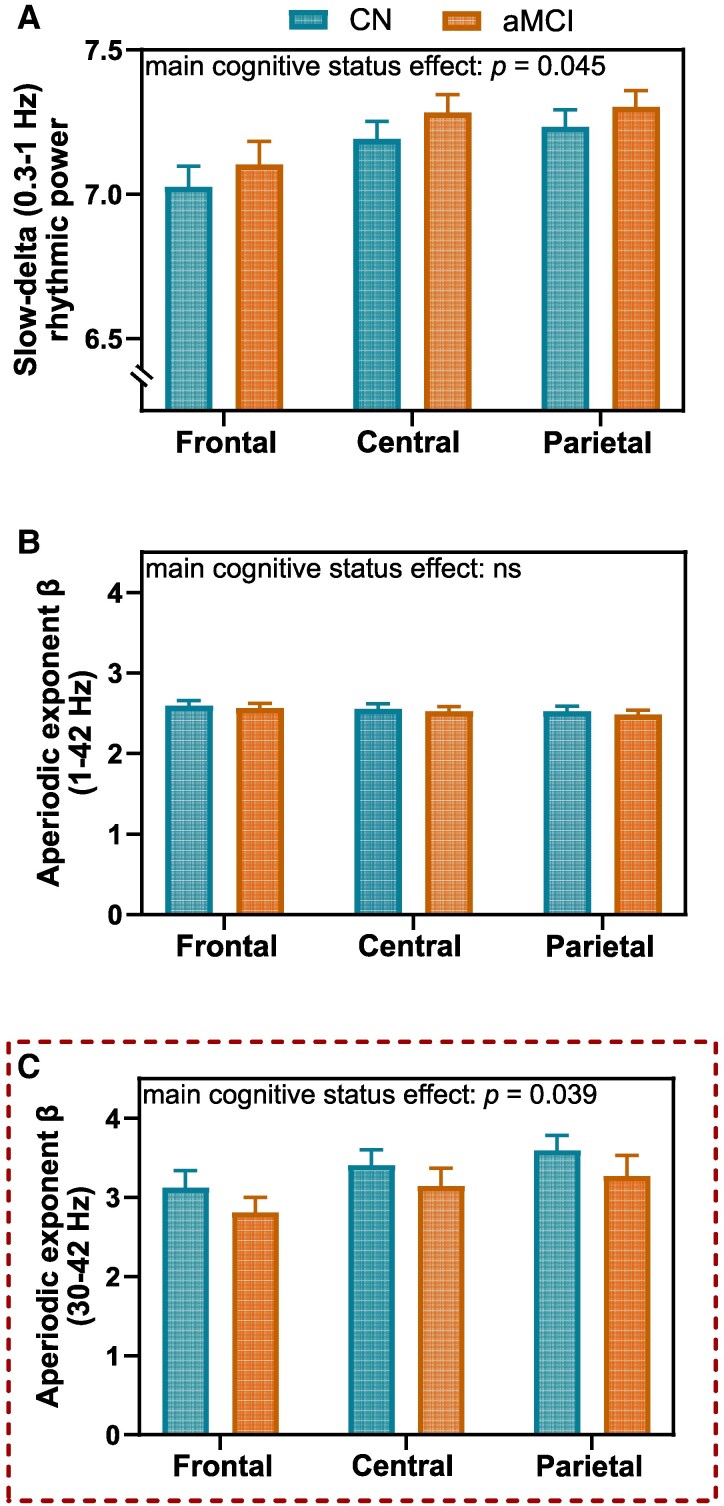
**Group comparisons of slow-delta rhythmic power and aperiodic exponents.** Panels show group means with 95% CIs (CN, *N* = 56; aMCI, *N* = 56). (**A**) Slow-delta rhythmic power (0.3–1 Hz) showed a significant main effect of cognitive status [two-way mixed ANOVA: *F*(1,110) = 4.13; *P* = 0.045; η^2^*p* = 0.036], with higher values in aMCI, and a significant effect of topography (*P* < 0.001), but no interaction. (**B**) Broadband aperiodic exponent (1–42 Hz) showed no main effect of cognitive status [*F*(1,110) = 0.60; *P* = 0.44; η^2^*p* = 0.005] and no interaction, but a significant topography effect (*P* < 0.001). (**C**) Gamma aperiodic exponent (30–42 Hz) showed a significant main effect of cognitive status [*F*(1,110) = 4.39; *P* = 0.039; η^2^*p* = 0.038], reflecting lower values in aMCI, and a significant topography effect (*P* < 0.001), with no interaction. The dashed dark red rectangle highlights effects that remained significant after accounting for EEG system effects. CN, cognitively normal; aMCI, amnestic mild cognitive impairment.

Finally, using a two-way mixed ANOVA, we separately analysed the rhythmic power in the slow-delta range (0.3–1 Hz). There was no statistically significant interaction between the cognitive status and topography on slow-delta rhythmic power [*F*(1.93,211.89) = 0.15; *P* = 0.85; η^2^*p* = 0.001]. By contrast, the main effect of cognitive status showed a statistically significant difference in mean slow-delta rhythmic power [*F*(1,110) = 4.13; *P* = 0.045; η^2^*p* = 0.036]. The aMCI group had a significantly higher slow-delta rhythmic power level than the CN group (see Fig. 5A). A main significant effect of topography was also observed [*F*(1.93,211.89) = 51.01; *P* < 0.001; η^2^*p* = 0.32; frontal < (central = parietal)].

In sum, the aMCI group showed lower fast-sigma/slow-beta rhythmic power in frontal, central and parietal regions and higher left parietal gamma rhythmic power. Slow-delta rhythmic power was also higher in the aMCI group compared to the CN group.

### Arrhythmic components

For the broadband aperiodic exponent (1–42 Hz), no statistically significant interaction between the cognitive status and topography was observed [*F*(1.65,181.67) = 0.55; *P* = 0.55; η^2^*p* = 0.005]. Similarly, the main effect of cognitive status was statistically non-significant [*F*(1,110) = 0.60; *P* = 0.44; η^2^*p* = 0.005; see Fig. 5B). A main significant effect of topography was however found [*F*(1.65,181.67) = 60.43; *P* < 0.001; η^2^*p* = 0.36; frontal > (central > parietal)]. For the gamma aperiodic exponent (30–42 Hz), no statistically significant interaction between the cognitive status and topography was observed [*F*(1.20,131.98) = 0.36; *P* = 0.59; η^2^*p* = 0.003]. Conversely, the main effect of cognitive status showed a statistically significant difference in the mean values of the gamma aperiodic exponent between the two groups [*F*(1,110) = 4.39; *P* = 0.039; η^2^*p* = 0.038]. Compared to the CN group, the aMCI group presented significantly lower gamma aperiodic exponents, reflecting a flattened spectral slope in this frequency range (see Fig. 5C). At last, a main significant topography effect was also observed for the gamma aperiodic exponent [*F*(1.20, 131.98) = 82.10; *P* < 0.001; η^2^*p* = 0.43; (frontal < central) < parietal].

In sum, while broadband aperiodic exponent did not differ between groups, the aMCI group showed significantly lower gamma aperiodic exponents than the CN group, indicating a flattened spectral slope, independent of the topographical region.

### Testing the EEG system effects

This section examines whether the use of different EEG systems influenced the significant results reported above (see the Assessment of the EEG system effect subsection in the Materials and methods section). First and most importantly, we observed no interaction between the factors of the EEG system and cognitive status for all analyses below (for all details, see Supplementary material, Results section). For the remaining details of our verification analyses, we report our observations following the order of the previous sections.

For the absolute spectral power analyses (for details, see Supplementary Table 3), two-way ANOVAs showed a significant main effect of the EEG system for all clusters (all *P*s < 0.018), except for parietal theta/alpha clusters (*P* > 0.05). This suggests a persistent impact of the EEG system on absolute power analyses. However, this EEG system effect was independent of the cognitive status effect. Indeed, main cognitive status effects for frontal, central and parietal clusters extending the sigma/beta range (all *P*s < 0.018), and P3’s cluster in the theta/alpha range (*P* = 0.049), remained significant. This demonstrates robust between-group differences in these spectral intervals. Otherwise, the other theta/alpha clusters became non-significant when considering the EEG system effect (all *P*s > 0.05).

For the rhythmic spectral power analyses (for details, see Supplementary Table 4), two-way ANOVAs showed a significant main effect of the EEG system for every cluster (all *P*s < 0.004). This suggests a persistent influence of the EEG system on rhythmic power analyses. However, this EEG system effect was independent of the cognitive status effect for every cluster. Indeed, a main cognitive status effect for frontal, central and parietal clusters covering the fast-sigma/slow-beta range (all *P*s < 0.004) and P3’s cluster in the gamma range (*P* = 0.003) remained significant. This confirms a robust, significant cognitive status effect on NREM sleep rhythmic power in these frequency ranges. For the slow-delta rhythmic power analysis (for details, see Supplementary Table 5), a three-way mixed ANOVA showed a non-significant main effect of the EEG system (*P* = 0.17). The main cognitive status effect on the slow-delta rhythmic power became marginally significant (*P* = 0.072).

For the analysis of the broadband aperiodic exponent, a three-way mixed ANOVA showed non-significant main effects of the EEG system (*P* = 0.44) and cognitive status (*P* = 0.33), confirming previous conclusions. For the analysis of the gamma aperiodic exponent, a three-way mixed ANOVA highlighted a significant main effect of the EEG system (*P* = 0.033). The main cognitive status effect on the gamma aperiodic exponent remained statistically significant (*P* = 0.023), confirming a robust between-group difference. All details regarding these results can be found in Supplementary Table 5. As part of our verifications, we also conducted cluster-based permutation tests with data split according to the EEG system, for which all details are in the Supplementary material, Results section. In sum, while the EEG system had some main effects on absolute and rhythmic power measures, these effects were largely independent of the cognitive status effect, and all key between-group differences remained stable, demonstrating that the reported cognitive status effects are not driven by the EEG system used.

Consistent with these results, cluster-based permutation tests split according to the EEG system revealed highly reproducible spectral patterns and aligned peaks of maximal between-group differences for rhythmic power across EEG systems (Supplementary Figs. 7 and 8), despite reduced statistical power in split samples. In contrast, several findings from traditional absolute spectral analyses lost significance after accounting for EEG system effects (Supplementary Table 3) and showed greater variability across systems in these verification analyses (Supplementary Figs. 5 and 6). Together, these findings indicate that while the EEG system factor introduces main effects on spectral estimates, rhythmic/arrhythmic spectral decomposition yields group differences that are resilient to instrumentation-related variability.

### Relationships with cognitive performance

**Figure 6:**
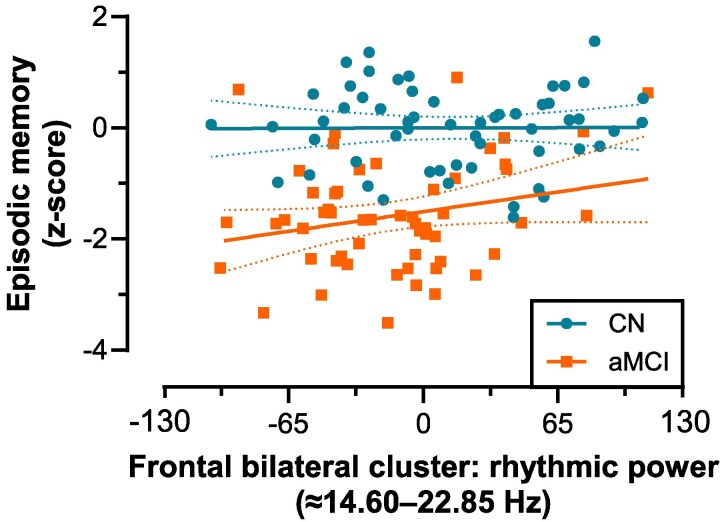
**Association between frontal fast-sigma/slow-beta rhythmic power and episodic memory differs by cognitive status.** Residualized scatterplots adjusted for EEG system effects are shown with 95% CIs represented by the dotted lines (CN, *N* = 56; aMCI, *N* = 56). Moderation analysis revealed a significant interaction [Δ*R*^2^ = 0.02; *F*(1,107) = 4.53; *P* = 0.036], such that higher rhythmic power predicted better memory performance in the aMCI group (*b* = 0.01; *t* = 2.69; *P* = 0.008), but not in CN participants [*b* = 0.00; *t* = −0.02; *P* = 0.98]. Each point represents an individual participant. CN, cognitively normal; aMCI, amnestic mild cognitive impairment.

Our exploratory moderation analyses examined rhythmic and arrhythmic activity variables that displayed group differences. We aimed to investigate their relationships with cognitive performance, specifically episodic and executive composite scores, and to determine whether cognitive status moderated these relationships while controlling for the effects of the EEG system. As shown in Table 2, across all models, cognitive status was significantly and negatively associated with episodic memory performance (all *P* < 0.001). None of the spectral measures (rhythmic or arrhythmic) demonstrated a significant association with episodic memory performance. However, a significant moderation effect by cognitive status was found only for frontal bilateral rhythmic power within the ∼14.60–22.85 Hz range, indicating that the relationship between frontal rhythmic power and episodic memory performance varied depending on cognitive status. The interaction term between the cognitive status and the frontal rhythmic power in the fast-sigma/slow-beta range induced a significant increase in explained variance [Δ*R^2^* = 0.02; *F*(1,107) = 4.53; *P* = 0.036; η^2^*p* = 0.04] of the episodic memory composite score. In the CN group, frontal bilateral rhythmic power did not show a significant association with episodic memory (*b* = 0.00; SE = 0.002; *t* = −0.02; *P* = 0.98; 95% confidence interval [CI] [−0.004, 0.004]). In contrast, as illustrated in Fig. 6, higher frontal bilateral rhythmic power was significantly linked to better episodic memory performance in the aMCI group (*b* = 0.01; SE = 0.002; *t* = 2.69; *P* = 0.008; 95% CI [0.002, 0.010]).

**Table 2:** Moderating effects of cognitive status on the association between spectral measures and episodic memory composite score (*Z*-score)

Predictors	*b*	SE	*t*	Sig.	95% CI
Frontal bilateral cluster: rhythmic power (≈14.60–22.85 Hz) (*X*)	0.00	0.002	−0.02	0.98	[−0.004, 0.004]
Cognitive status (*W*)	−1.43	0.15	−9.32	**<0.001**	[−1.74, −1.13]
Interaction *X* × *W*	0.01	0.003	2.13	**0.036**	[0.0004, −0.0117]
EEG system	−0.61	0.18	−3.33	**0.001**	[−0.96, −0.25]
Central bilateral cluster: rhythmic power (≈15.72–20.56 Hz) (*X*)	0.001	0.003	0.20	0.84	[−0.006, 0.007]
Cognitive status (*W*)	−1.46	0.16	−9.41	**<0.001**	[−1.77, −1.15]
Interaction *X* × *W*	0.006	0.005	1.14	0.26	[−0.004, 0.015]
EEG system	−0.69	0.18	−3.91	**<0.001**	[−1.04, −0.34]
Parietal bilateral cluster: rhythmic power (≈16.06–20.61 Hz) (*X*)	0.002	0.004	0.40	0.69	[−0.006, 0.009]
Cognitive status (*W*)	−1.45	0.16	−9.29	**<0.001**	[−1.76, −1.14]
Interaction *X* × *W*	0.005	0.005	0.89	0.37	[−0.006, 0.015]
EEG system	−0.68	0.18	−3.78	**<0.001**	[−1.03, −0.32]
Parietal unilateral cluster: rhythmic power (≈30.71–41.36 Hz) (*X*)^a^	−0.004	0.003	−1.25	0.21	[−0.009, 0.002]
Cognitive status (*W*)	−1.42	0.16	−9.15	**<0.001**	[−1.73, −1.12]
Interaction *X* × *W*	−0.002	0.004	0.42	0.68	[−0.009, 0.006]
EEG system	−0.67	0.17	−3.93	**<0.001**	[−1.01, −0.33]
Frontal aperiodic exponent (30–42 Hz) (*X*)	0.11	0.13	0.81	0.42	[−0.16, 0.37]
Cognitive status (*W*)	−1.47	0.15	−9.63	**<0.001**	[−1.77, −1.17]
Interaction *X* × *W*	0.10	0.20	0.49	0.62	[−0.30, 0.50]
EEG system	−0.73	0.17	−4.29	**<0.001**	[−1.06, −0.39]
Central aperiodic exponent (30–42 Hz) (*X*)	0.13	0.15	0.92	0.36	[−0.16, 0.43]
Cognitive status (*W*)	−1.48	0.15	−9.76	**<0.001**	[−1.78, −1.18]
Interaction *X* × *W*	0.03	0.20	0.14	0.89	[−0.36, 0.42]
EEG system	−0.73	0.17	−4.34	**<0.001**	[−1.07, −0.40]
Parietal aperiodic exponent (30–42 Hz) (*X*)	0.19	0.15	1.30	0.20	[−0.10, 0.50]
Cognitive status (*W*)	−1.47	0.15	−9.65	**<0.001**	[−1.77, −1.17]
Interaction *X* × *W*	−0.07	0.19	−0.35	0.72	[−0.43, 0.30]
EEG system	−0.73	0.17	−4.35	**<0.001**	[−1.07, −0.40]

*Notes.* Bold-coloured results identify significant results at *P*  *<* 0.05. *N* = 112, unless stated otherwise.

*b*, unstandardized regression coefficient; SE, standard error; CI, confidence interval.

^a^Missing data for one participant with aMCI.

Across all models predicting executive function composite score, cognitive status was significantly associated with poorer outcomes (all *P* < 0.001), whereas neither rhythmic power nor aperiodic exponent measures showed significant associations with executive function performance. No significant interactions were observed between spectral measures and cognitive status (all *P*s > 0.39).

## Discussion

The spectral analysis, differentiating the rhythmic and arrhythmic activity, revealed that the aMCI group had reduced rhythmic power in the fast-sigma/slow-beta range across all examined regions. In contrast, there was an increase in power in the gamma frequency range, but this was restricted to the left parietal area. Additionally, inter-individual differences in frontal fast-sigma/slow-beta rhythmic power predicted episodic memory performance within the aMCI group only. Moreover, the mean values of the gamma aperiodic exponent were significantly lower across all examined regions in the aMCI group compared to the CN group. This indicates a flattening of the gamma spectral slope in the aMCI group. Finally, no significant group differences were found for delta rhythmic power or the global aperiodic exponent.

### Absolute power spectral analysis during non-rapid eye movement sleep

In this study, we first undertook to replicate previous cognitive status effects on the traditional spectral measure of absolute power. Mostly consistent with previous studies^41-43^ and with our predictions, we found that the aMCI group showed lower power in the theta/alpha (limited to the left parietal area) and sigma/beta ranges (across all topographies) compared to the CN group. However, no previous study found significant differences in the beta range for total night-time NREM sleep.^41,42^ This difference could result from separating the frequency spectrum into canonical bands in previous studies, which could have weakened the magnitude of the effect in the beta range. Another contributing factor may be the heterogeneity among groups regarding cognitive impairments (e.g. subjective cognitive impairment and various subtypes of MCI) in those studies. Infirming our prediction, no significant difference was found in the delta spectral range (1–4 Hz), as in some previous studies.^41,44,45^ However, the impact of EEG arrhythmic activity on absolute power estimates was not addressed in those studies, which may have contributed to the variability observed in this limited literature.

### Cognitive status effects on rhythmic and arrhythmic brain activity

Using the more refined analysis of rhythmic/arrhythmic spectral components, we found significantly lower rhythmic power in the aMCI group in the fast-sigma/slow-beta range across all topographies investigated, mostly supporting our predictions. This suggests an early alteration of thalamo-cortical networks during NREM sleep in aMCI. Neuropathological processes affecting the thalamus in MCI (i.e. atrophy and neuroinflammation) may account for this oscillatory deficit. Indeed, a large recent study (*N* = 632) reported a modest but significant positive association between fast spindle density (central frequency: 15.5 Hz) and thalamic volume.^91^ Another large study (*N* = 1386) showed that thalamic volume reduction is an early marker of neurodegeneration observed in MCI.^92^ Moreover, a meta-analysis reported elevated levels of neuroinflammation biomarkers (i.e. 18 kDa translocator protein) in the thalamus of individuals with MCI compared with healthy controls, among several other regions.^93^ Consistent with this line of evidence, a recent study^35^ showed, in CN older adults with Alzheimer’s disease–related risk factors, that microglial activation (i.e. neural cells mediating CNS inflammatory processes) was associated with increased tau levels and synaptic degeneration, in turn predicting age-related reductions in fast-sigma (13–16 Hz) rhythmic power over frontocentral regions. Lower fast-sigma power was further associated with poorer overnight memory consolidation. Additionally, slow-sigma and beta absolute power levels, mostly covering the frontocentral area, were negatively associated with tau and synaptic integrity markers and positively correlated with overnight memory consolidation. This aligns with the association we observed specifically between frontal rhythmic power in the fast-sigma/slow-beta range and episodic memory performance in the aMCI group and is consistent with prior literature highlighting the frontal sensitivity of sleep electrophysiological markers to neurocognitive integrity in ageing.^29,30,94-96^ Based on the potential contribution of fast spindle events on fast-sigma/slow-beta rhythmic power, the decrease in power we observed is coherent with findings from event-based analyses in the existing literature. Specifically, two studies reported a significant reduction in the quantity of fast spindles in aMCI groups compared to controls.^43,97^ Altogether, these findings support the view that oscillatory alterations in the fast-sigma/slow-beta range could reflect early pathophysiological processes linking thalamo-cortical dysfunction, neuroinflammation and cognitive decline.

Furthermore, the aMCI group showed unanticipated higher gamma rhythmic power limited to the left parietal area. Gamma activity during sleep is usually considered to index cortical arousal.^98^ Indeed, health conditions characterized by ‘hyperarousal’, such as insomnia and post-traumatic stress disorder, were found to lead to higher gamma power during NREM sleep.^99,100^ Therefore, a possible explanation is that aMCI, through underlying pathological processes, would increase cortical arousal during NREM sleep. Although aMCI is a heterogeneous clinical condition and not all individuals necessarily progress to Alzheimer’s disease,^101^ several animal models of Alzheimer’s disease support the observation of higher gamma power during NREM sleep.^102-106^ One can hypothesize that alterations in rhythmic gamma activity during NREM sleep could represent an early marker of Alzheimer’s disease–related pathological changes. Gamma oscillations during the wake–sleep cycle arise from the contributions of excitatory and inhibitory neurons, with a greater implication from the latter.^107^ It was proposed that the increased coherence of those oscillations during NREM sleep might be an ideal property to support sleep-dependent memory processes. Disruptions of E–I balance, as reported in Alzheimer’s disease,^60^ could therefore lead to aberrantly increased gamma oscillatory activity during NREM sleep, potentially impairing memory-related processing. Consistent with the central role of the parietal cortex in memory formation,^108,109^ we observed higher parietal gamma rhythmic power in aMCI. Notably, this effect appears to track overall group-level amnestic status rather than inter-individual variability in episodic memory. This suggests that gamma alterations during NREM sleep may be less sensitive to subtle cognitive variation, become more predictive later in the Alzheimer’s disease continuum or reflect more fine-grained pathophysiological changes.

Infirming our prediction, we did not find robust between-group differences regarding delta rhythmic power. Nevertheless, we observed weak differences in the slow-delta range, where the aMCI group presented higher power than the CN group. Analogously, a previous study showed a similar tendency where participants with long-life cognitive decline displayed higher slow-delta power than participants with cognitive improvement.^110^ A large study involving older adults (*N* = 3819) also reported that higher slow-delta power during NREM sleep was robustly associated with worse cognitive performance.^16^ Moreover, we previously observed a significant increase in the proportion of SWs with a slow negative-to-positive-phase transition in aMCI participants relative to CN controls.^69^ When considering all SW together, this translated as a significant slowing of this transition in aMCI. Djonlagic *et al*.^16^ observed that the increased duration of SW events (0.5–2 Hz) strongly correlated with increased slow-delta power (<1 Hz) within the delta range. In the ageing context, the slow-delta power might thus be an indirect measure of a finer underlying electrophysiological process, namely the prolongation of SW events. Consequently, event-based analyses may be a more direct and sensitive approach than spectral analysis to probe changes in the delta range during ageing.

As predicted, the aMCI group showed a significant decrease in gamma aperiodic exponent mean values in all areas compared to the CN group, indicating a flattening of the gamma spectral slope. According to current neurophysiological models,^53,54,111,112^ this change suggests increased neuronal excitability and alterations of background neural activity in participants with aMCI during NREM sleep. This interpretation is reinforced by reports of neuronal network hyperexcitability observed early in the Alzheimer’s disease spectrum,^61-63^ particularly during NREM sleep in the form of subclinical epileptiform activity.^113,114^ Additionally, a recent study revealed an association between flattened spectral slopes in the gamma range (30–48 Hz) during sleep (non-stage-specific) and increased subsequent levels of plasma Aβ, as measured through all-night blood sampling, in healthy adults aged 20–68 years.^115^ Thus, one could hypothesize that the flattening of the gamma spectral slope during sleep could be linked to higher central production of Aβ. It could also reflect increased cerebral clearance of Aβ, which could act as a compensatory mechanism in individuals with aMCI. Supporting a link with Alzheimer’s disease–related pathophysiology, a resting-state MEG study^116^ revealed that the aperiodic spectral slope (15–50 Hz) was significantly flattened in patients with Alzheimer’s disease relative to controls. This change in aperiodic activity was associated with greater cognitive impairment and correlated with both amyloid-β and tau burden. A similar pattern has been reported in prodromal Alzheimer’s disease patients, who displayed a reduced gamma-band aperiodic exponent (30–45 Hz) over posterior regions during resting-state EEG compared to CN individuals, regardless of their amyloid status (Aβ+ or Aβ−).^117^ These resting-state findings suggest that flattening of the gamma-range aperiodic spectral slope reflects an early alteration in background neural excitability along the continuum of Alzheimer’s disease. Our results further support this idea by demonstrating that similar alterations occur during NREM sleep, a brain state characterized by diminished sensory input and tightly regulated E–I dynamics.^118^ This suggests that these changes may have functional significance for sleep-dependent processes.

An alternative interpretation of our findings can be made by examining the connection between the gamma spectral slope and cortical arousal levels. This slope was shown to get steeper from wakefulness to NREM sleep and REM sleep.^56^ Consequently, a flattened slope during NREM sleep may indicate a shift towards a more wake-like level of brain arousal. This observation aligns with the consistently shallower sleep seen in MCI,^40^ including in the current study, and the gamma rhythmic power increase we observed. Together, these findings suggest that gamma aperiodic alterations during NREM sleep in aMCI may reflect the combined influence of heightened neuronal excitability, altered sleep depth and early Alzheimer’s disease–related neurobiological changes. Future research could benefit from directly comparing the methods used in this study with other spectral decomposition techniques (e.g. FOOOF and IRASA) applied to the same sleep datasets. This would help clarify their relative sensitivity and how they might complement one another in assessing sleep-related neurophysiological changes.

Contrary to our initial hypothesis, the broadband aperiodic exponent did not show any differences between the groups in the topographical regions we investigated. This unexpected result aligns with recent evidence that the low- and high-frequency ranges can reflect distinct aperiodic components rather than a single scale-free process.^119^ Future research using high-density EEG is essential to refine the topographical characterization of NREM rhythmic and arrhythmic electrophysiological changes beyond the regions we examined.

### Methodological considerations

Traditional absolute spectral power reflects a complex mixture of rhythmic (oscillatory) and arrhythmic (scale-free) neural activity, whereas rhythmic/arrhythmic spectral decomposition isolates oscillatory power after accounting for the underlying aperiodic component. In this context, the persistence of sigma-beta power group differences across both approaches suggests robust oscillatory alterations, whereas divergences at lower (theta/alpha) and higher (gamma) frequencies indicate that effects previously reported in these ranges^42,43^ may partly reflect changes in background aperiodic activity rather than rhythmic power *per se*. Accounting for the aperiodic component therefore refines the physiological interpretation of spectral alterations, particularly at the extremes of the frequency spectrum.

Neuroimaging research increasingly relies on multicentric collaborations, data sharing and pooled datasets.^120^ However, this approach requires careful consideration of instrumentation-related variability, particularly in the EEG subfield. In the present study, EEG data were acquired using different recording systems, and the impact of the EEG system was explicitly evaluated across all primary analyses. Both traditional absolute power and rhythmic/arrhythmic analyses showed significant main effects of the EEG system. Critically, however, group differences derived from rhythmic/arrhythmic spectral decomposition remained largely significant after statistically controlling for the EEG system effects using parametric statistical models (ANOVAs), with the exception of slow-delta rhythmic power (Supplementary Tables 4 and 5). Consistent with these results, cluster-based permutation tests split according to the EEG system revealed highly reproducible rhythmic spectral patterns across EEG systems, whereas absolute spectral measures showed greater variability and reduced robustness. These findings indicate that rhythmic/arrhythmic decomposition produces group differences that are more resilient to instrumentation-related variability, supporting its use in multicentric EEG studies.

### Limitations

Some limitations of our study should be addressed. First, the National Institute on Aging and Alzheimer’s Association’s (NIA-AA) research framework proposed biological definitions of the different stages across the Alzheimer’s disease spectrum.^121^ In this study, we instead applied a syndromic definition of aMCI. Therefore, we could not ensure that Alzheimer’s disease caused the memory impairments of our participants with aMCI. In the absence of biomarker confirmation, the present interpretations should be viewed as hypothesis generating. Additionally, some of the aMCI criteria were evaluated differently across the protocols. However, all criteria were fulfilled by validated means, which can have ecological value as MCI is assessed by diverse tools in clinics. Future studies should confirm our results in participants with aMCI presenting an Alzheimer’s disease–related biomarker profile and investigate whether similar EEG changes can be observed in cognitively unimpaired individuals who are positive for Alzheimer’s disease biomarkers. Second, no formal *a priori* power analysis was conducted, as sample size was constrained by the availability of eligible participants and strict matching criteria. Although this may limit sensitivity to detect small group differences, our sample size was comparable to or larger than that of most prior sleep EEG studies in aMCI and was sufficient to detect the primary effects reported. Third, participants recruited later across the four protocols were recorded with the Natus system (System 2) and were likely to be more cognitively impaired, as some were recruited from a memory clinic rather than the community. As a result, controlling for the EEG system may have partially accounted for variance related to cognitive status, potentially attenuating associations between spectral measures and cognitive performance. This may explain why the EEG system covariate was consistently associated with the episodic memory score across moderation models. Finally, estimating rhythmic activity in the slow-delta range (0.3–1 Hz) presents significant methodological challenges. Frequencies close to the high-pass filter cut-off at 0.3 Hz may be affected by the characteristics of the filter and its transition bandwidths, which were not fully specified for the recording systems used. While no significant EEG system effects on slow-delta rhythmic power were noted, these results should be interpreted with caution and confirmed in future studies using recording systems with well-characterized low-frequency filter properties.

## Conclusion

In conclusion, our new method for EEG spectral analysis demonstrates greater robustness compared to the traditional spectral analysis method. Our findings indicate a loss of fast-sigma/slow-beta oscillatory activity and signs of cortical hyperexcitability during NREM sleep in individuals with aMCI. These results are consistent with recent findings in the literature. Our results thus demonstrate the sensitivity of NREM sleep as a window on early brain changes in individuals at higher risk of progressing to Alzheimer’s disease. Our study may have therapeutic implications as well. Evidence suggests that non-invasive brain stimulation^122-124^ and pharmacological approaches^125,126^ can modulate NREM brain activity, including enhancing sigma power. Future studies should determine whether such interventions could restore NREM sleep rhythmic and arrhythmic activity and mitigate cognitive decline in older adults at higher Alzheimer’s disease risk.

## Supplementary Material

fcag204_Supplementary_Data

## Data Availability

Data used in the present study will be available from the corresponding author upon request. All custom code used for data preprocessing and analysis in this study is publicly available on GitHub at https://github.com/AlexLaf11/eeg-nrem-amci.git
