# Supplementary material for: Altered rhythmic and arrhythmic electroencephalographic activity during non-rapid eye movement sleep in amnestic mild cognitive impairment
Source: Brain Commun. 2026 Jun 22;8(3):fcag204. doi: 10.1093/braincomms/fcag204 (PMC13284422; doi:10.1093/braincomms/fcag204)
Supplement: fcag204_Supplementary_Data [file fcag204_supplementary_data.docx]

**Altered rhythmic and arrhythmic electroencephalographic activity during non-REM sleep in amnestic mild cognitive impairment**

**Short title**: Non-REM sleep EEG spectral alterations in aMCI

**Authors**

Alexandre Lafrenière ^1,2*^, Jean-Marc Lina ^1,3,4^, Claire André ^1,2^, Marie-Ève Martineau-Dussault ^1,2^, Dominique Lorrain ^5,6^, Célyne Bastien ^7,8^, Carol Hudon ^7,8^, Nadia Gosselin ^1,2^ & Julie Carrier ^1,2*^

^1^Center for Advanced Research in Sleep Medicine, Hôpital du Sacré-Cœur de Montréal, CIUSSS du Nord-de-l'Île-de-Montréal, Montreal, Canada

^2^Department of Psychology, Université de Montréal, Montreal, Canada

^3^Department of Electrical Engineering, École de Technologie Supérieure, Montreal, Canada

^4^Centre de Recherches Mathématiques, Université de Montréal, Montreal, Canada

^5^Research Centre on Aging, University Institute of Geriatrics of Sherbrooke, CIUSSS de l'Estrie - CHUS, Sherbrooke, Canada

^6^Department of Psychology, Université de Sherbrooke, Sherbrooke, Canada

^7^CERVO Research Centre, Quebec City, Canada

^8^School of Psychology, Université Laval, Quebec City, Canada

**Corresponding authors**

Alexandre Lafrenière*

Julie Carrier*

Center for Advanced Research in Sleep Medicine, Hôpital du Sacré-Cœur de Montréal, CIUSSS du Nord-de-l'Île-de-Montréal,

5400 Gouin West Boulevard, Montreal, Quebec, H4J 1C5,

Canada.

alexandre.lafreniere.2@umontreal.ca

julie.carrier.1@umontreal.ca

**METHODS**

**Calculation of the cognitive composite scores**

1. Each participant's raw performance on the episodic memory and executive function measures was z-transformed using the means and standard deviations of the CN participants.
2. Before the next step, some z-scores were reversed when appropriate so that their value follows the same logic of interpretation as the other z-scores.
3. Finally, all domain-specific z-scores were averaged, creating measures of each participant's global performance in episodic memory and executive functions.

Verbal episodic learning and memory measures used for the memory score:

- Rey Auditory Verbal Learning Test (RAVLT) ^1^: Sum of trials 1 to 5, List B, Immediate recall, Delayed recall.
- Logical Memory Stories ^2^: Immediate recall, Delayed recall.

Executive function measures used for the executive function score:

- Trail Making Test (TMT) ^3^: Contrast Part B - Part A (time).
- D-KEFS - Stroop test ^4^: Contrast Part 4 – Parts 1+2 (time), Contrast Part 3 – Part 1 (time), Part 3: Total errors.

For the CN group, one participant lacked the D-KEFS - Stroop test. For the aMCI group, one participant lacked the measure of RAVLT-Delayed recall, another one lacked both measures of the Logical Memory Stories, and two participants lacked either the complete D-KEFS - Stroop test or a subtest. Thus, their composite scores were computed based on the remaining available tests or subtests.

**Statistical analysis: assessment of the EEG system effect**

As our data were recorded with two different EEG systems, our set of verifications implicated the conduction of cluster-based permutation tests split according to the EEG system. This aimed to verify if the same pattern of differences was found between our groups, regardless of the EEG system used. Because these verification analyses involved a data split, leading to decreased statistical power, we tested two sensitivity levels when identifying the spectral clusters. As for the original analyses, we started by using a cluster forming threshold of a *t*-value of ± 2 (corresponding to the critical value of a bilateral *t*-test at alpha-level 0.05 in the *t*-distribution). Then, we increased the sensitivity for cluster identification to overcome the reduced statistical power. This was achieved by *a priori* reducing the cluster-forming threshold to ± 1.5.

Moreover, given that the data split could influence the matching of the groups, we compared both groups on sociodemographic variables to determine whether significant differences arose. For the analyses involving System 1 (CN: *n* = 42; aMCI: *n* = 37), the groups did not differ on any sociodemographic variable (all *p*s > .18). For System 2 (CN: *n* = 14; aMCI: *n* = 19), both groups were significantly different (*t*(31) = -2.68, *p* = .012) relative to the variable of age (CN: 71.79 ± 4.96 years old; aMCI: 76.68 ± 5.34 years old). No other differences were found (all *p*s > .15). Consequently, in a second step, the significant clusters identified were compared between both groups using an ANCOVA controlling for the effect of age. This ensured that any group differences were not imputable to an age effect. All analyses involving System 2 specifically controlled for this age effect.

**Supplementary Table 1.** Neuropsychological tests used to assess the cognitive status

| **Cognitive domain** | **Core cognitive tests** | **Additional cognitive tests** |
| --- | --- | --- |
| **Attention & processing speed** | D-KEFS - Stroop test ^4^:   - *Part 1 (time)* - *Part 2 (time)*   WAIS-III or IV – Coding ^5,6^  Trail Making Test ^3^: *Part A (time)* | CPT ^7^:   - *Omission errors* - *Variability in reaction time*   Digit span forward ^5^: *maximum*  Bells test ^8^: *omissions* |
| **Executive functions** | Trail Making Test ^3^: *Part B - Part A (time)*  D-KEFS - Stroop test ^4^:   - *Part 3 – Part 1 (time)* - *Part 4- Part 3 (time)* | Tower of London ^9^   - *Total number of movements* - *Total time*   CPT ^7^: *commission errors*  Digit span backwards ^5^: *maximum.* |
| **Verbal and visual episodic learning and memory** | Rey Auditory Verbal Learning Test ^1^:   - *Sum of trials 1 to 5* - *Immediate recall* - *Delayed recall*   Logical Memory Stories ^2^ (WMS-III or Rouleau version):   - *Immediate recall* - *Delayed recall* | Brief Visual Memory Test-Revised ^10^:   - *Total recall (trials 1 to 3)* - *Delayed recall*   Rey-Osterrieth Complex Figure ^11^:   - *Immediate recall* - *Delayed recall* |
| **Visuospatial abilities** | Rey-Osterrieth Complex Figure ^11^: *copy score* | Benton Line Orientation Judgment ^12^  WAIS-III - Blocks design ^5^ |
| **Language** | Boston Naming Test - 30 items version ^13^: *number of correct answers*  Verbal fluency ^4^: *semantic (number of words)* | WAIS-III – Vocabulary ^5^  Verbal fluency ^4^: *phonemic* *(number of words)* |
| **Global cognitive functioning: screening tests** | MoCA* ^14^ | MMSE ^15^ |

CPT, Continuous Performance Test; D-KEFS, Delis-Kaplan Executive Function System; MMSE, Mini Mental State Examination; MoCA, Montreal Cognitive Assessment; WAIS $[III or IV]$, Wechsler Adult Intelligence Scale; WMS, Wechsler Memory Scale.

*Note*. Core neuropsychological tests were available for all participants for every cognitive domain, with certain additional tests differing between protocols. All available cognitive tests were used to establish the participants’ cognitive status.

^*^ Missing data in 6 participants (*n* = 2 CN, *n* = 4 aMCI).

**Supplementary Table 2.** Criteria for the aMCI diagnosis across protocols

| **Criteria** | **Method for measurement** | **Notes** |
| --- | --- | --- |
| 1) Subjective cognitive complaints | A. Cognitive Failures Questionnaire ^16^ (three protocols; *n* = 75) | Subjective complaint established according to Villeneuve et al.’s criteria ^17^:  i) Total score > 24;  ii) Or the response 3 (“*quite often*”) or 4 (“*very often*”) on at least one item. |
|  | B. Cognitive Complaint Questionnaire ^18^ (one protocol; *n* = 37) | i) Total score ≥ 3.  ii) Or specific item criteria. |
| 2) Objective cognitive impairment | All available neuropsychological tests per domain | See Supplementary Table 1 |
| 3) Preservation of independence in daily activities | A. Activities of Daily Living Inventory ^19^  (three protocols; *n* = 90) | i) Each aMCI participant's raw score was z-transformed using the means and SD of the CN participants.  ii) Participants at ≤ 2 SD were then further investigated to determine the potential presence of dementia.  iii) The participants were either included or excluded based on the nature of their low score and the degree of cognitive impairment. |
|  | B. Structured interview (one protocol; *n* = 22) | Participants were interrogated on whether they have had, in the last weeks, any difficulties in completing daily tasks (i.e., cooking, cleaning, running errands, driving, managing finances, taking medications), which they were able to complete previously, due to cognitive problems. |
| 4) Absence of dementia or other causes for cognitive impairment | Based on criterion 3 and the medical history obtained during the screening and interview |  |

**Supplementary**
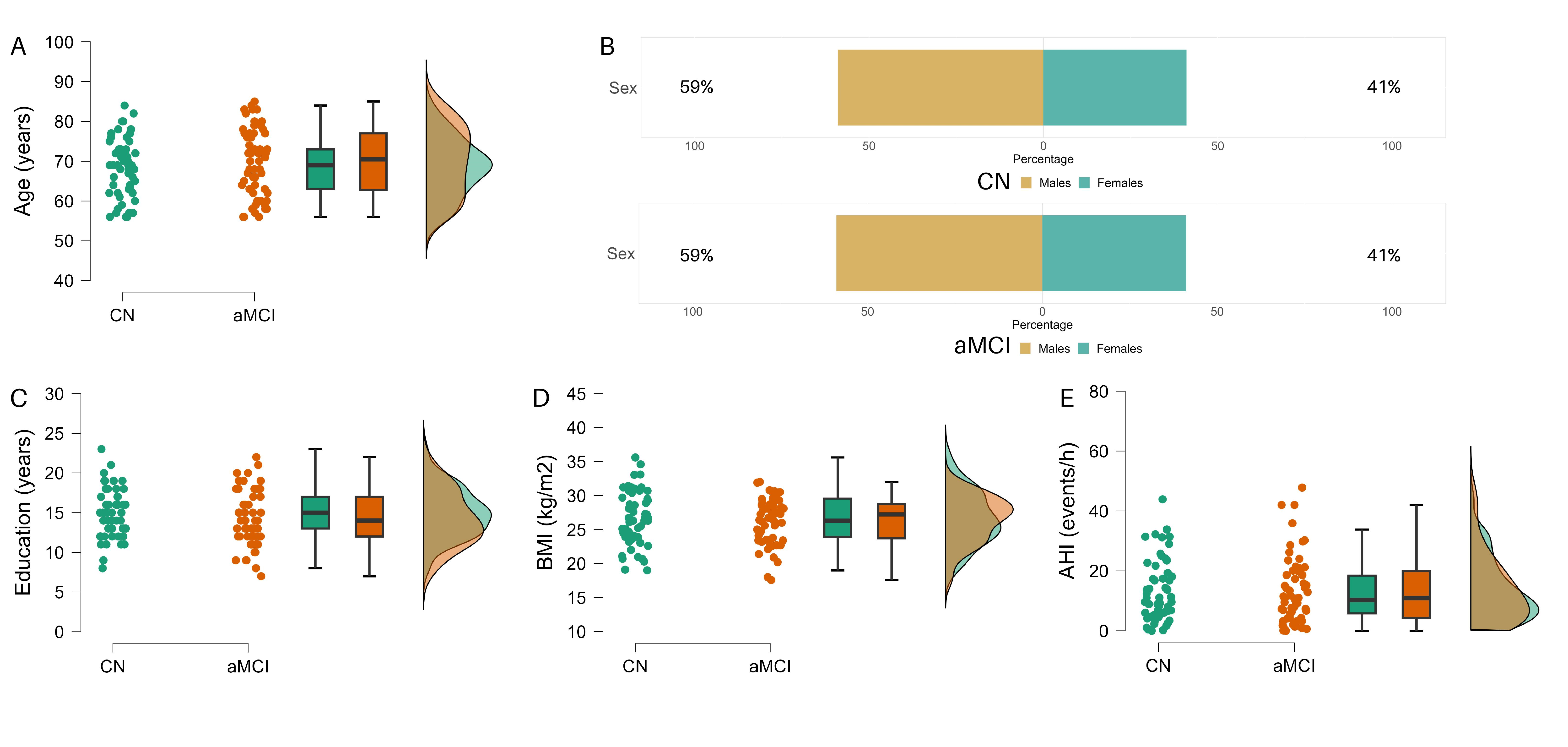
**Figure 1. Descriptive plots displaying the distribution of the matched variables in each group**. **A, C, D, E**. Left: a cloud of points illustrating each variable value per group (CN, *N* = 56; aMCI, *N* = 56). Middle: box plots with thick dark lines representing the median and the boxes depicting the interquartile ranges. Right: one-sided violin plots presenting the smoothed distributions of the data. **B**. Likert plot displaying the proportion of biological sexes in both groups. CN, cognitively normal; aMCI, amnestic mild cognitive impairment; BMI, body mass index; AHI, apnea-hypopnea index.

**
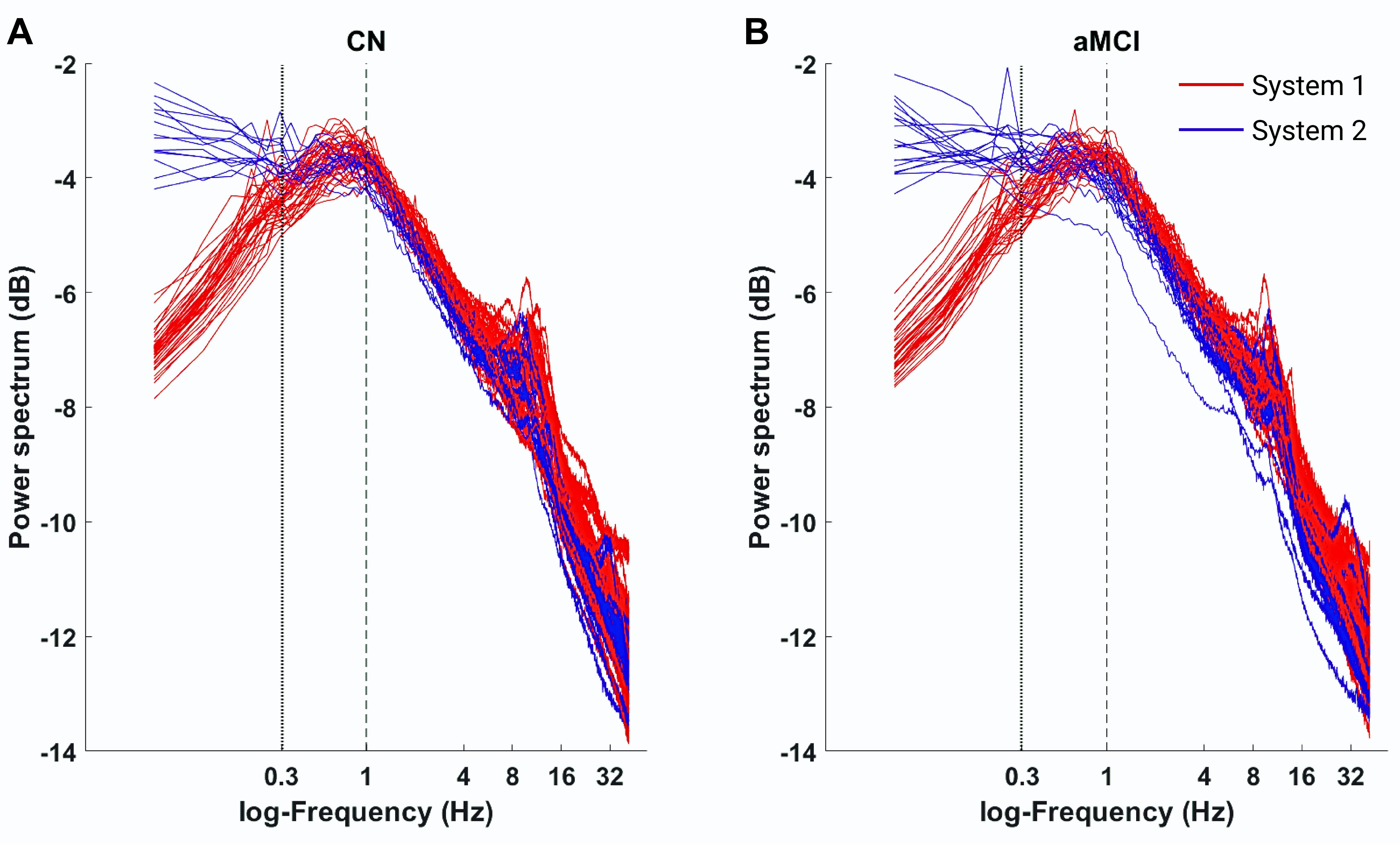
**

**Supplementary Figure 2. Power spectra stratified by EEG recording system and cognitive status group.** Each line represents the power spectrum of an individual participant; Panel A shows CN participants and Panel B shows aMCI participants. Recordings acquired with the Grass system are shown in red (*N* = 79), and those acquired with the Natus system in blue (*N* = 33). System-related differences are primarily observed in the very low-frequency range (<1 Hz), whereas spectral profiles above 1 Hz are largely comparable. CN = cognitively normal, aMCI = amnestic mild cognitive impairment, dB = decibels, Hz = hertz.

**
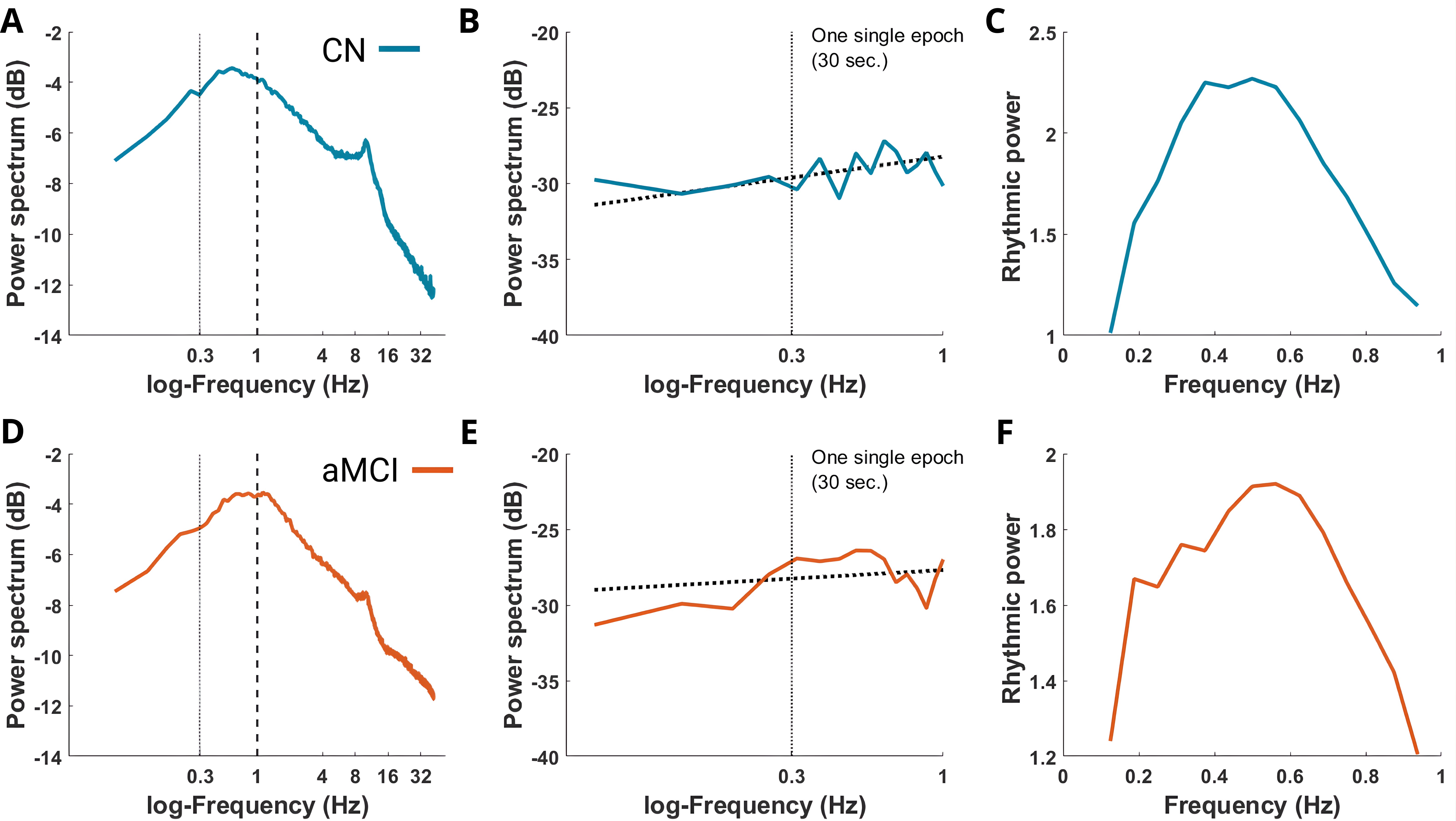
**

**Supplementary Figure 3. Illustration of slow-delta rhythmic power (0.3-1 Hz) in each cognitive status group.** Left panels: Power spectra from one representative participant per group (A = CN, *N* = 1; D = aMCI, *N* = 1). Middle panels: Fitted spectral slope in the 0.3-1 Hz range used to estimate residual rhythmic power within a single NREM epoch (B = CN, *N* = 1; E = aMCI, *N* = 1). Right panels: Slow-delta rhythmic power averaged across multiple artifact-free epochs for the same participants (C = CN, *N* = 1; F = aMCI, *N* = 1). CN = cognitively normal, aMCI = amnestic mild cognitive impairment, dB = decibels, Hz = hertz.

**
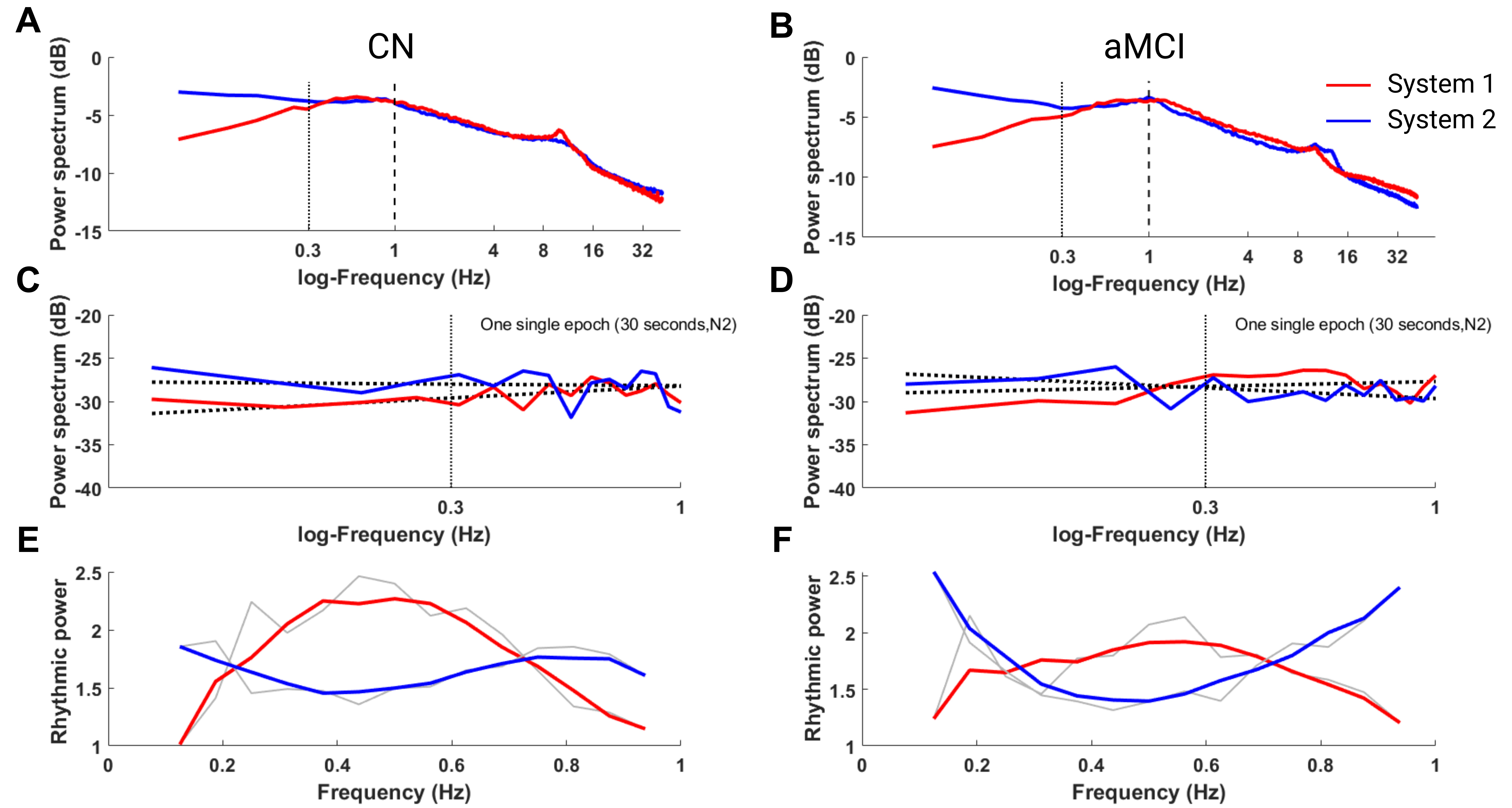
**

**Supplementary Figure 4. Illustration of slow-delta rhythmic power (0.3-1 Hz) across cognitive groups and EEG recording systems.** Top row: Power spectra from two representative participants per group recorded with different EEG systems (A = CN, *N* = 2; B = aMCI, *N* = 2). Middle row: Fitted spectral slopes in the 0.3-1 Hz range used to estimate residual rhythmic power within a single epoch, accounting for system-specific “spectral plateau” tilt (C = CN, *N* = 2; D = aMCI, *N* = 2). Bottom row: Slow-delta rhythmic power averaged over a set of artifact-free epochs for the same participants (E = CN, *N* = 2; F = aMCI, *N* = 2). Despite system-related differences in low-frequency “spectral plateau” tilt, comparable levels of rhythmic power are obtained across systems (N.B., the y-axis is zoomed-in). Red lines indicate recordings acquired with the Grass system; blue lines indicate recordings acquired with the Natus system. CN, cognitively normal; aMCI, amnestic mild cognitive impairment; dB, decibels; Hz, hertz.

**RESULTS**

**Absolute spectral power analysis**

**Supplementary Table 3.** Group comparisons of absolute spectral power clusters (integrated power, i.e., the area under the curve derived from the significant clusters) considering the EEG system effect.

|  | ANOVA | |  | |  |  |
| --- | --- | --- | --- | --- | --- | --- |
| Clusters (C) | Cognitive status | Effect (η^2^*p*) | EEG system | Effect (η^2^*p*) | Interaction | Effect (η^2^*p*) |
| **Frontal: F3** |  |  |  |  |  |  |
| C1: *6.25-9.38 Hz* | *F* = 1.53, *p* = .22 |  | ***F* = 5.78, *p* = .018** | S1 < S2 (.051) | *F* = 0.59, *p* = .44 |  |
| C2: *11.38-26.07 Hz* | ***F* = 7.11, *p* = .009** | CN > aMCI (.062) | ***F* = 27.24, *p* < .001** | S1 > S2 (.20) | *F* = 0.00, *p* = .95 |  |
| **Frontal: F4** |  |  |  |  |  |  |
| C1: *5.37-9.57 Hz* | *F* = 1.30, *p* = .26 |  | ***F* = 5.93, *p* = .017** | S1 < S2 (.052) | *F* = 0.27, *p* = .60 |  |
| C2: *11.57-27.59 Hz* | ***F* = 5.80, *p* = .018** | CN > aMCI (.051) | ***F* = 27.71, *p* < .001** | S1 > S2 (.20) | *F* = 0.04, *p* = .85 |  |
| **Central: C3** |  |  |  |  |  |  |
| C1: *13.67-21.44 Hz* | ***F* = 9.03, *p* = .003** | CN > aMCI (.077) | ***F* = 12.63, *p* < .001** | S1 > S2 (.11) | *F* = 1.92, *p* = .17 |  |
| **Central: C4** |  |  |  |  |  |  |
| C1: *13.23-21.88 Hz* | ***F* = 7.80, *p* = .006** | CN > aMCI (.067) | ***F* = 15.19, *p* < .001** | S1 > S2 (.12) | *F* = 1.34, *p* = .25 |  |
| **Parietal: P3** ^a^ |  |  |  |  |  |  |
| C1: *7.76-10.01 Hz* | ***F* = 3.96, *p* = .049** | CN > aMCI (.036) | *F* = 0.95, *p* = .33 |  | *F* = 0.45, *p* = .51 |  |
| C2: *13.57-18.21 Hz* | ***F* = 10.62, *p* = .001** | CN > aMCI (.090) | ***F* = 8.10, *p* = .005** | S1 > S2 (.070) | *F* = 1.95, *p* = .17 |  |
| **Parietal: P4** |  |  |  |  |  |  |
| C1: *8.94-10.84 Hz* | *F* = 1.69, *p* = .20 |  | *F* = 0.08, *p* = .78 |  | *F* = 0.04, *p* = .85 |  |
| C2: *13.57-22.80 Hz* | ***F* = 12.18, *p* < .001** | CN > aMCI (.10) | ***F* = 22.16, *p* < .001** | S1 > S2 (.17) | *F* = 2.89, *p* = .092 |  |

*Note.* The variables were subjected to a ln transformation. Bold-colored results identify significant results at *p* <.05.

^a^ Missing data in one participant with aMCI due to persistent artifacts on P3 (CN: *n* = 56; aMCI: *n* = 55).

**Rhythmic and arrhythmic spectral power analysis**

**Supplementary Table 4.** Group comparisons of rhythmic spectral power clusters (integrated power, i.e., the area under the curve derived from the significant clusters) considering the EEG system effect.

|  | ANOVA | |  | |  |  |
| --- | --- | --- | --- | --- | --- | --- |
| Clusters (C) | Cognitive status | Effect (η^2^*p*) | EEG system | Effect (η^2^*p*) | Interaction | Effect (η^2^*p*) |
| **Frontal: F3** |  |  |  |  |  |  |
| C1: *14.16 - 22.85 Hz* | ***F* = 10.44, *p* = .002** | CN > aMCI (.088) | ***F* = 27.06, *p* < .001** | S1 > S2 (.20) | *F* = 0.22, *p* = .64 |  |
| **Frontal: F4** |  |  |  |  |  |  |
| C1: *14.21 - 20.85 Hz* | ***F* = 9.18, *p* = .003** | CN > aMCI (.078) | ***F* = 26.01, *p* < .001** | S1 > S2 (.19) | *F* = 0.34, *p* = .56 |  |
| **Central: C3** |  |  |  |  |  |  |
| C1: *15.82 - 20.56 Hz* | ***F* = 9.29, *p* = .003** | CN > aMCI (.079) | ***F* = 12.23, *p* < .001** | S1 > S2 (.10) | *F* = 0.78, *p* = .38 |  |
| **Central: C4** |  |  |  |  |  |  |
| C1: *15.72 - 20.17 Hz* | ***F* = 8.50, *p* = .004** | CN > aMCI (.073) | ***F* = 14.50, *p* < .001** | S1 > S2 (.12) | *F* = 0.73, *p* = .39 |  |
| **Parietal: P3** ^a^ |  |  |  |  |  |  |
| C1: *16.06 - 20.61 Hz* | ***F* = 10.94, *p* = .001** | CN > aMCI (.093) | ***F* = 13.84, *p* < .001** | S1 > S2 (.12) | *F* = 1.16, *p* = .28 |  |
| C2: *30.71 - 41.36 Hz* | ***F* = 9.53, *p* = .003** | CN < aMCI (.082) | ***F* = 8.81, *p* = .004** | S1 < S2 (.076) | *F* = 0.42, *p* = .52 |  |
| **Parietal: P4** |  |  |  |  |  |  |
| C1: *16.06 - 20.17 Hz* | ***F* = 10.79, *p* = .001** | CN > aMCI (.091) | ***F* = 20.51, *p* < .001** | S1 > S2 (.16) | *F* = 1.25, *p* = .27 |  |

Bold-colored results identify significant results at *p* <.05.

^a^ Missing data in one participant with aMCI due to persistent artifacts on P3 (CN: *n* = 56; aMCI: *n* = 55).

**Supplementary Table 5.** Group comparisons of the aperiodic exponents and the slow-delta rhythmic power when considering the EEG system effect.

|  | ANOVA | |  | |  |  |  |
| --- | --- | --- | --- | --- | --- | --- | --- |
| Variables | **A**: Cognitive status | Effect (η^2^*p*) | **B**: EEG system | Effect (η^2^*p*) | **C**: Topography | Effect (η^2^*p*)^a^ | Interactions |
| **Rhythmic spectral power** |  |  |  |  |  |  |  |
| *Slow-delta (0.3-1 Hz)* | *F* = 3.31, *p* = .072 |  | *F* = 1.95, *p* = .17 |  | ***F* = 40.92, *p* < .001** | F < [C = P] (.28) | *p* > .05 |
| **Aperiodic exponents** |  |  |  |  |  |  |  |
| *Broadband (1-42 Hz)* | *F* = 0.94, *p* = .33 |  | *F* = 0.62, *p* = .44 |  | ***F* = 53.32, *p* < .001** | F > [C > P] (.33) | *p* > .05 |
| *Gamma (30-42 Hz)* | ***F* = 5.29, *p* = .023** | CN > aMCI (.047) | ***F* = 4.69, *p* = .033** | S1 > S2 (.042) | ***F* = 64.63, *p* < .001** | [F < C] < P (.37) | *p* > .05 |

Bold-colored results identify significant results at *p* <.05.

^a^ All topographical differences presented as the symbols > or < are significant at *p < .05* after a Bonferroni adjustment for multiple comparisons.


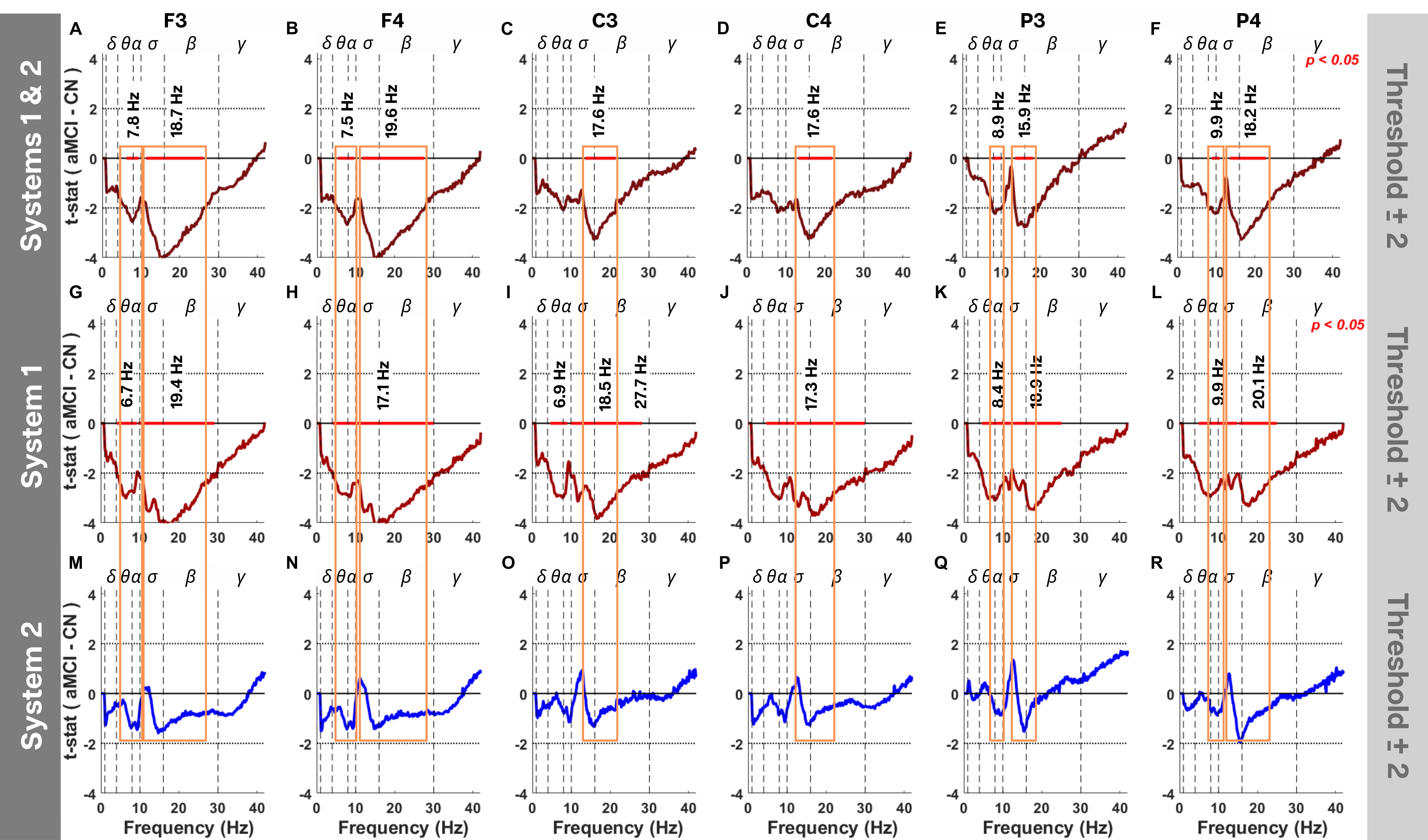


**Supplementary Figure 5**. **Between-group comparisons of absolute spectral power using cluster-based permutation testing, stratified by EEG recording system with the standard cluster-forming threshold (±2).**Top row (A-F): Plots of frequency-specific *t*-values from the main group comparison across topographies (CN, *N* = 56; aMCI, *N* = 56). Middle row (G-L): Plots of frequency-specific *t*-values from between-group comparisons across topographies for EEG System 1 (CN, *N* = 42; aMCI, *N* = 37). Bottom row (M-R): Plots of frequency-specific *t*-values from between-group comparisons across topographies for EEG System 2 (CN, *N* = 14; aMCI, *N* = 19) . Thick red lines represent the frequency range covered by the significant clusters, with numbers above denoting cluster median frequencies. Orange rectangles highlight the alignments of ‘peaks of maximal *t*-values’ between analyses, with the significant clusters of the main comparison as reference. CN = cognitively normal, aMCI = amnestic mild cognitive impairment, Hz = hertz.


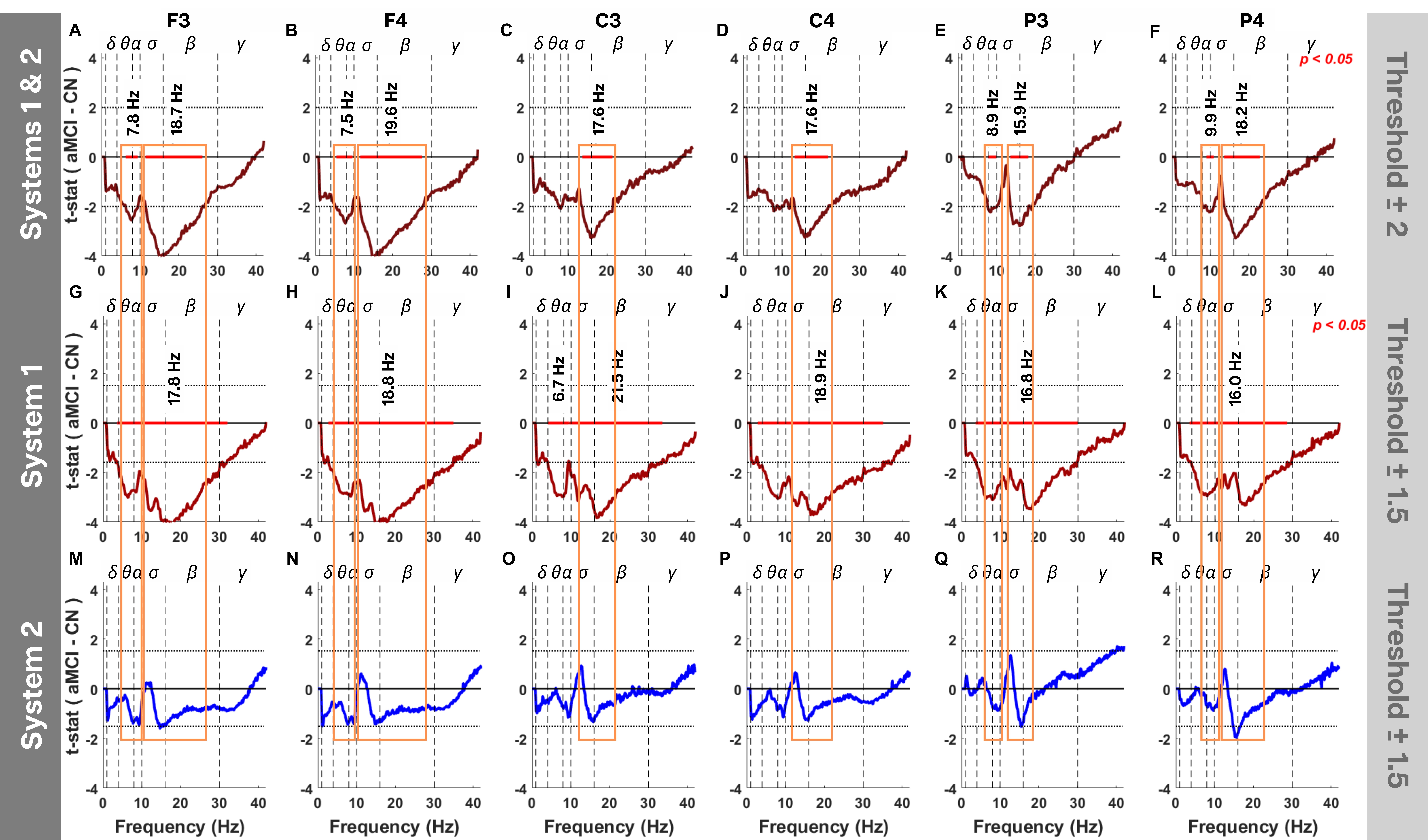


**Supplementary Figure 6.** **Between-group comparisons of absolute spectral power using cluster-based permutation testing, stratified by EEG recording system with a more sensitive cluster-forming threshold**. Top row (A-F): Plots of frequency-specific *t*-values from the main group comparison across topographies using the standard cluster-forming threshold (±2) (CN, *N* = 56; aMCI, *N* = 56). Middle row (G-L): Plots of frequency-specific *t*-values from between-group comparisons across topographies for EEG System 1 using a more sensitive cluster-forming threshold (±1.5) (CN, *N* = 42; aMCI, *N* = 37). Bottom row (M-R): Plots of frequency-specific *t*-values from between-group comparisons across topographies for EEG System 2 using a more sensitive cluster-forming threshold (±1.5) (CN, *N* = 14; aMCI, *N* = 19). Thick red lines represent the frequency range covered by significant clusters, with numbers above denoting cluster median frequencies. Orange rectangles highlight the alignment of ‘peaks of maximal *t*-values’ across analyses, using the significant clusters of the main comparison as reference. CN, cognitively normal; aMCI, amnestic mild cognitive impairment; Hz, hertz.

General comment: Supplementary Figures 5 and 6 illustrate analyses split according to the EEG system, each applying a different sensitivity level for cluster identification. Three main observations emerged from this set of verifications. First, regardless of the cluster-forming threshold applied for the analyses involving System 1, the significant clusters extended greater frequency ranges than the main comparison (combining data from Systems 1 and 2). The aMCI group had lower absolute power in most of the spectrum compared to the CN group (see Supplementary Figures 5 and 6). Second, despite sensitivity level adjustments for cluster identification, no significant clusters were identified for the analyses involving System 2. Third, when looking at Supplementary Figures 5 and 6, one interesting observation is that all ‘peaks of maximal *t*-values’ (i.e., spectral regions of maximal between-group differences) are replicated from the main analysis (top row) to the analyses split according to the EEG systems (middle and bottom rows). These ‘peaks of maximal *t*-values’ translated as significant clusters in the main analysis and the analyses involving EEG System 1, and as non-significant in analyses involving EEG System 2. This latter result might be due to a lack of statistical power resulting from the smaller sample sizes of each group (CN: *n* = 14; aMCI: *n* = 19).

**
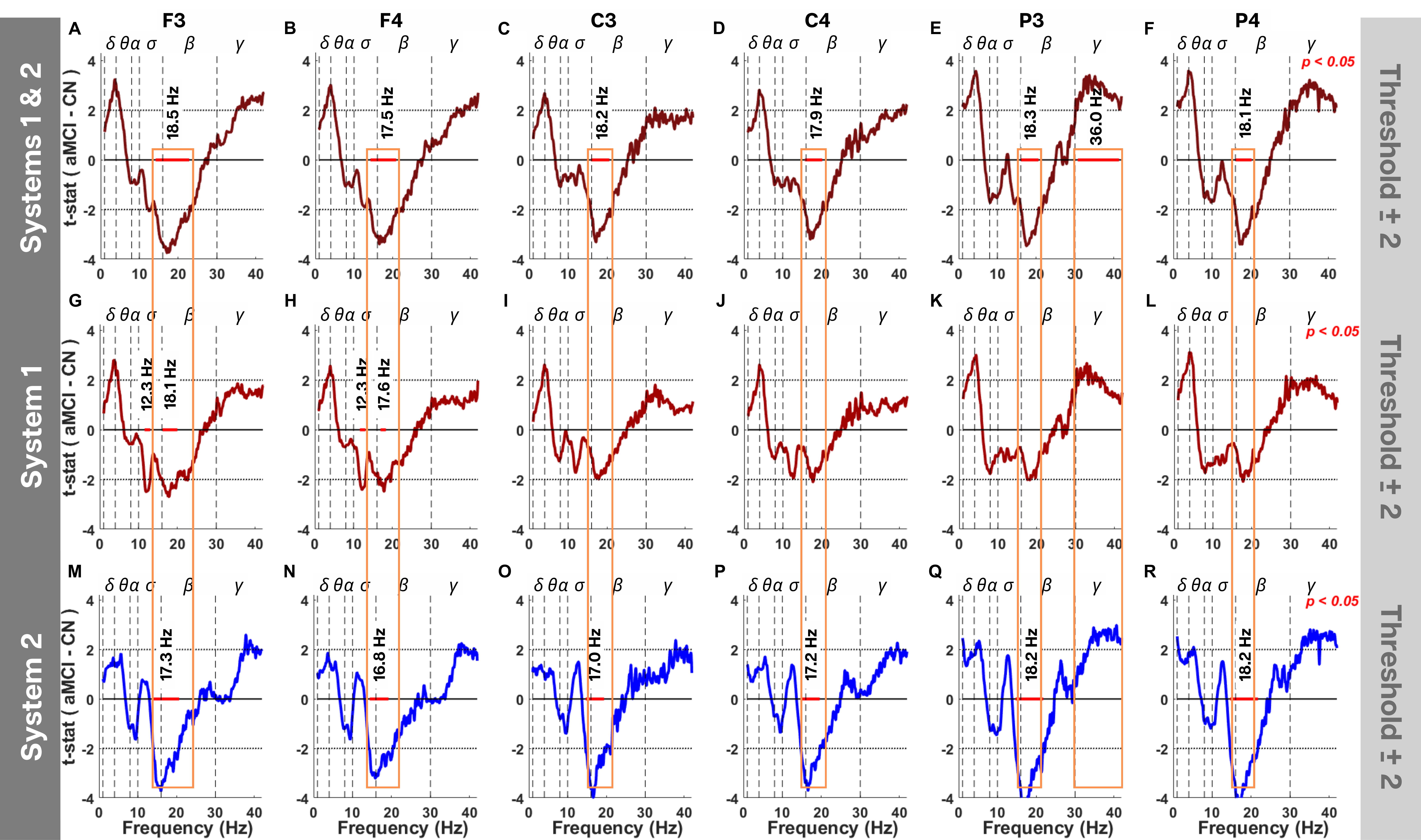
**

**Supplementary Figure 7**. **Between-group comparisons of rhythmic spectral power using cluster-based permutation testing, stratified by EEG recording system with the standard cluster-forming threshold (±2).** Top row (A-F): Plots of frequency-specific *t*-values from the main group comparison across topographies (CN, *N* = 56; aMCI, *N* = 56). Middle row (G-L): Plots of frequency-specific *t*-values from between-group comparisons across topographies for EEG System 1 (CN, *N* = 42; aMCI, *N* = 37). Bottom row (M-R): Plots of frequency-specific *t*-values from between-group comparisons across topographies for EEG System 2 (CN, *N* = 14; aMCI, *N* = 19) . Thick red lines represent the frequency range covered by the significant clusters, with numbers above denoting cluster median frequencies. Orange rectangles highlight the alignments of ‘peaks of maximal *t*-values’ between analyses, with the significant clusters of the main comparison as reference. CN = cognitively normal, aMCI = amnestic mild cognitive impairment, Hz = hertz.

**
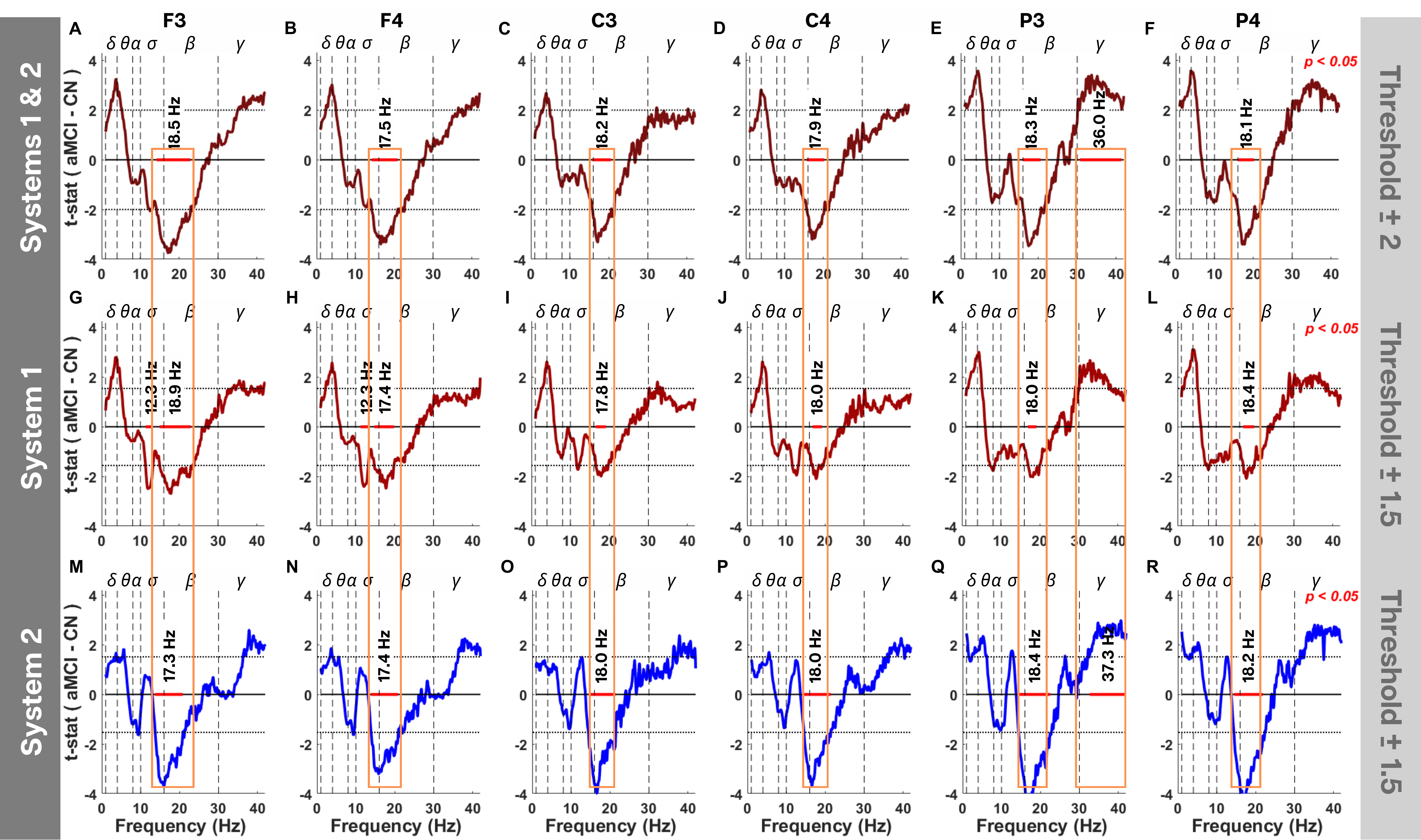
Supplementary Figure 8.** **Between-group comparisons of rhythmic spectral power using cluster-based permutation testing, stratified by EEG recording system with a more sensitive cluster-forming threshold**. Top row (A-F): Plots of frequency-specific *t*-values from the main group comparison across topographies using the standard cluster-forming threshold (±2) (CN, *N* = 56; aMCI, *N* = 56). Middle row (G-L): Plots of frequency-specific *t*-values from between-group comparisons across topographies for EEG System 1 using a more sensitive cluster-forming threshold (±1.5) (CN, *N* = 42; aMCI, *N* = 37). Bottom row (M-R): Plots of frequency-specific *t*-values from between-group comparisons across topographies for EEG System 2 using a more sensitive cluster-forming threshold (±1.5) (CN, *N* = 14; aMCI, *N* = 19). Thick red lines represent the frequency range covered by significant clusters, with numbers above denoting cluster median frequencies. Orange rectangles highlight the alignment of ‘peaks of maximal *t*-values’ across analyses, using the significant clusters of the main comparison as reference. CN, cognitively normal; aMCI, amnestic mild cognitive impairment; Hz, hertz.

General comment: First, for the analyses involving System 1, an adjustment of sensitivity level for cluster identification (±1.5) allowed the re-detection of all main analysis’ significant clusters extending the sigma/slow-beta spectral range. The only exception was for the cluster covering the gamma range, which was nonetheless marginally significant (P3: 30.08 - 37.65 Hz, *p* = .068; CN < aMCI). All clusters from the main analysis were thus re-identified by overcoming the statistical power reduction (resulting from splitting the data) by slightly increasing the sensitivity of the cluster-forming threshold. Second, every cluster in the sigma/slow-beta spectral range identified in the main analysis was replicated for the analyses involving System 2, regardless of the cluster-forming threshold applied. The only exception was for the cluster extending the gamma range, which went from marginally significant (P3: 33.69 - 41.99 Hz, *p* = .051; CN < aMCI) to significant depending on the sensitivity level of the cluster-forming threshold (±2 versus ±1.5). Using ANCOVAs, the main effect of the cognitive status on rhythmic power in all these clusters remained significant when controlling for the age effect (threshold at ± 2: all *p*s < .009; threshold at ± 1.5: all *p*s < .045). Third, when looking at Supplementary Figures 7 and 8, we can observe that all ‘peaks of maximal *t*-values’ (i.e., spectral regions of maximal between-group differences) are replicated from the main analysis (top row) to the analyses split according to the EEG systems (middle and bottom rows). As a consequence, clusters of rhythmic power differences identified in the main analysis were, to a great extent of precision, replicated in the analyses split according to the EEG system. This supports the robustness of the cognitive status effect on NREM sleep rhythmic power. Additionally, the stability of rhythmic power differences throughout analyses split according to the EEG system suggests greater robustness of this analysis method than traditional absolute power analyses. Moreover, these findings demonstrate that our results are stable and not dependent on the specific cluster-forming threshold selected.

**System 1 – Slow-delta rhythmic power (0.3-1 Hz).** In a two-way mixed ANOVA, no statistically significant interaction was observed between the cognitive status and topography on slow-delta rhythmic power (*F*(1.76, 135.64) = 0.17, *p* = .82, η^2^*p* = .002). The main effect of cognitive status was also statistically non-significant for mean slow-delta rhythmic power (*F*(1, 77) = 1.79, *p* = .18, η^2^*p* = .023). Qualitatively, the aMCI group (*x̄_aMCI_* = 7.21, *SEM* = 0.036) showed the same trend of a higher slow-delta rhythmic power level than the CN group (*x̄_CN_* = 7.14, *SEM* = 0.034). Finally, a main significant effect of topography was observed (*F*(1.76, 135.64) = 40.27, *p* < .001, η^2^*p* = .34; Frontal < [Central = Parietal]).

**System 2 – Slow-delta rhythmic power (0.3-1 Hz).** In a two-way mixed ANCOVA controlling for the age effect, no statistically significant interaction was observed between the cognitive status and topography on slow-delta rhythmic power (*F*(2, 60) = 0.30, *p* = .74, η^2^*p* = .010). The main effect of cognitive status was also statistically non-significant for mean slow-delta rhythmic power (*F*(1, 30) = 2.03, *p* = .17, η^2^*p* = .063). Qualitatively, the aMCI group (*x̄_aMCI_* = 7.27, *SEM* = 0.040) showed the same trend of a higher slow-delta rhythmic power level than the CN group (*x̄_CN_* = 7.18, *SEM* = 0.047). At last, the main effect of topography was non-significant (*F*(2, 60) = 1.12, *p* = .34, η^2^*p* = .036).

**Arrhythmic components**

**System 1 – Aperiodic exponents.**

*Broadband (1-42 Hz)*: In a two-way mixed ANOVA, no statistically significant interaction was found between the cognitive status and topography (*F*(1.51, 116.21) = 1.92, *p* = .16, η^2^*p* = .024). Likewise, the main effect of cognitive status was statistically non-significant (*F*(1, 77) = 0.016, *p* = .90, η^2^*p* = .000). However, a main significant effect of topography was found (*F*(1.51, 116.21) = 59.13, *p* < .001, η^2^*p* = .43; Frontal > [Central > Parietal]).

*Gamma (30-42 Hz)*: In a two-way mixed ANOVA, no statistically significant interaction was observed between the cognitive status and topography (*F*(1.17, 89.86) = 0.51, *p* = .50, η^2^*p* = .007). Similarly, the main effect of cognitive status was also non-significant (*F*(1, 77) = 0.62, *p* = .43, η^2^*p* = .008). Qualitatively, the aMCI group (*x̄_aMCI_* = 3.26, *SEM* = 0.14) showed the same trend of a lower gamma aperiodic exponent than the CN group (*x̄_CN_* = 3.41, *SEM* = 0.13). Finally, a main significant effect of topography was found (*F*(1.17, 89.86) = 70.32, *p* < .001, η^2^*p* = .48; [Frontal < Central] < Parietal).

**System 2 – Aperiodic exponents.**

*Broadband (1-42 Hz)*: In a two-way mixed ANCOVA controlling for the age effect, no statistically significant interaction was found between the cognitive status and topography (*F*(1.83, 55.02) = 0.08, *p* = .91, η^2^*p* = .003). The main effect of cognitive status was also statistically non-significant (*F*(1, 30) = 0.47, *p* = .50, η^2^*p* = .015). Similarly, the main effect of topography was non-significant (*F*(1.83, 55.02) = 0.47, *p* = .61, η^2^*p* = .016).

*Gamma (30-42 Hz)*: In a two-way mixed ANCOVA controlling for the age effect, no statistically significant interaction was found between the cognitive status and topography (*F*(1.21, 36.17) = 0.25, *p* = .67, η^2^*p* = .008). Conversely, the main effect of cognitive status was statistically significant (*F*(1, 30) = 8.68, *p* = .006, η^2^*p* = .22). The aMCI group (*x̄_aMCI_* = 2.72, *SEM* = 0.12) showed a significant decrease in the gamma aperiodic exponent compared to the CN group (*x̄_CN_* = 3.27, *SEM* = 0.14). At last, the main topography effect was non-significant (*F*(1.21, 36.17) = 0.71, *p* = .43, η^2^*p* = .023).

General comment: Following data splitting, the same pattern of results tends to be reproduced for both exponents. Notwithstanding, the main effect of cognitive status on the gamma aperiodic exponent was only statistically significant in the sub-analysis involving System 2 (CN > aMCI); however, although non-significant, the same trend was observed when examining the groups’ mean for sub-analysis involving System 1 (CN > aMCI). This suggests a similar direction of difference between CN and aMCI groups, regardless of the dataset.

**References**

1. Rey A. L'examen psychologique dans les cas d'encéphalopathie traumatique.(Les problems.). *Arch Psychol (Geneve)*. 1941;

2. Wechsler D. *Wechsler Memory Scale-Third Edition*. The psychological corporation; 1997.

3. Reitan RM. Validity of the Trail Making Test as an indicator of organic brain damage. *Percept Mot Skills*. 1958;8(3):271-276.

4. Delis DC, Kaplan E, Kramer JH. Delis-Kaplan executive function system. 2001;

5. Wechsler D. *WAIS-III : administration and scoring manual : Wechsler Adult Intelligence Scale*. Psychological Corporation; 1997.

6. Wechsler D. *WAIS-IV administration and scoring manual*. Psychological Corporation; 2008.

7. Conners CK. *Conners’ Continuous Performance Test (CPT–II) Computer Program for Windows: Technical Guide and Software Manual*. Multi-Health Systems; 2000.

8. Gauthier L, Dehaut F, Joanette Y. The Bells Test: A quantitative and qualitative test for visual neglect. *International Journal of Clinical Neuropsychology*. 1989;11(2):49-54.

9. Culbertson WC, Zillmer EA. *Tower of London-Drexel University – 2nd Edition (TOLDX-2)*. MHS – Multi-Health Systems Inc.; 2005.

10. Benedict RHB. *Brief visuospatial memory test--revised*. PAR; 1997.

11. Osterrieth PA. Le test de copie d'une figure complexe: contribution à l'étude de la perception et de la mémoire. [Test of copying a complex figure; contribution to the study of perception and memory.]. *Arch Psychol (Geneve)*. 1944;30:206-356.

12. Qualls CE, Bliwise NG, Stringer AY. Short Forms of The Benton Judgment of Line Orientation Test: Development and Psychometric Properties. *Arch Clin Neuropsychol*. 2000;15(2):159-163.

13. Kaplan E, Goodglass H, Weintraub S. *Boston naming test*. Lea & Febiger; 1983.

14. Nasreddine ZS, Phillips NA, Bédirian V, et al. The Montreal Cognitive Assessment, MoCA: A Brief Screening Tool For Mild Cognitive Impairment. 2005;53(4):695-699. doi:<https://doi.org/10.1111/j.1532-5415.2005.53221.x>

15. Folstein MF, Folstein SE, McHugh PR. “Mini-mental state”: a practical method for grading the cognitive state of patients for the clinician. *J Psychiatr Res*. 1975;12(3):189-198.

16. Broadbent DE, Cooper PF, FitzGerald P, Parkes KR. The cognitive failures questionnaire (CFQ) and its correlates. *Br J Clin Psychol*. 1982;21(1):1-16.

17. Villeneuve S, Pepin V, Rahayel S, et al. Mild cognitive impairment in moderate to severe COPD: a preliminary study. *Chest*. 2012;142(6):1516-1523.

18. Antérion CT, Ribas C, Honoré-Masson S, Berne G, Ruel JH, Laurent B. Le questionnaire de plainte cognitive (QPC): un outil de recherche de plainte suspecte d'évoquer une maladie d'Alzheimer? *L'Année gérontologique*. 2003;17:56-65.

19. Galasko D, Bennett D, Sano M, et al. An inventory to assess activities of daily living for clinical trials in Alzheimer's disease. *Alzheimer Dis Assoc Disord*. 1997;11:33-39.
